# MicroRNAs Associated with Caste Determination and Differentiation in a Primitively Eusocial Insect

**DOI:** 10.1038/srep45674

**Published:** 2017-03-31

**Authors:** David H. Collins, Irina Mohorianu, Matthew Beckers, Vincent Moulton, Tamas Dalmay, Andrew F. G. Bourke

**Affiliations:** 1 School of Biological Sciences, University of East Anglia, Norwich Research Park, Norwich NR4 7TJ, UK; 2 School of Computing Sciences, University of East Anglia, Norwich Research Park, Norwich NR4 7TJ, UK

## Abstract

In eusocial Hymenoptera (ants, bees and wasps), queen and worker adult castes typically arise via environmental influences. A fundamental challenge is to understand how a single genome can thereby produce alternative phenotypes. A powerful approach is to compare the molecular basis of caste determination and differentiation along the evolutionary trajectory between primitively and advanced eusocial species, which have, respectively, relatively undifferentiated and strongly differentiated adult castes. In the advanced eusocial honeybee, *Apis mellifera*, studies suggest that microRNAs (miRNAs) play an important role in the molecular basis of caste determination and differentiation. To investigate how miRNAs affect caste in eusocial evolution, we used deep sequencing and Northern blots to isolate caste-associated miRNAs in the primitively eusocial bumblebee *Bombus terrestris*. We found that the miRNAs Bte-miR-6001-5p and -3p are more highly expressed in queen- than in worker-destined late-instar larvae. These are the first caste-associated miRNAs from outside advanced eusocial Hymenoptera, so providing evidence for caste-associated miRNAs occurring relatively early in eusocial evolution. Moreover, we found little evidence that miRNAs previously shown to be associated with caste in *A. mellifera* were differentially expressed across caste pathways in *B. terrestris*, suggesting that, in eusocial evolution, the caste-associated role of individual miRNAs is not conserved.

Eusocial insects are characterized by exhibiting castes, with the queen caste being specialized for reproduction and the worker caste being specialized for non-reproductive, colony tasks^1^,^2^,^3^. The occurrence of castes represents a leading example of polyphenism, where multiple adult phenotypes develop from the same genotype as a result of environmental influences during development^4^,^5^. Explaining the molecular basis of polyphenism represents a fundamental challenge in biology. The eusocial Hymenoptera (ants, bees and wasps) are the largest and most diverse group of eusocial insects^1^. Accordingly, to address the molecular basis of caste development, several previous studies have used genomic approaches such as high-throughput sequencing to profile the genes associated with caste determination and differentiation in a range of eusocial Hymenoptera^6^,^7^,^8^,^9^,^10^,^11^,^12^,^13^,^14^.

While these studies have been highly successful at isolating protein-coding genes associated with caste determination and differentiation, the role of microRNAs (miRNAs) in the same processes has been comparatively understudied. MiRNAs are a class of regulatory short RNAs (21–23 bp) that target mRNAs following transcription and prevent them from being translated into proteins^15^,^16^. They have been shown to have essential roles in development, metabolism, and other processes in animals^17^. Recent research has also shown that miRNAs are associated with polyphenisms in non-social insects, such as winged and wingless morphs in the pea aphid, *Acyrthosiphon pisum*^18^, and that they regulate the transition between solitary and gregarious morphs in the locust *Locusta migratoria*^19^. In eusocial Hymenoptera, the potential role of miRNAs in caste development has been studied in the honeybee, *Apis mellifera*, where miRNAs have been shown to be differentially expressed in queen- and worker-destined larvae and pupae^20^,^21^,^22^ and experimentally implicated in caste determination^23^.

*A. mellifera* is a well-studied example of the advanced eusocial Hymenoptera, which are typified by queens and workers that are morphologically strongly differentiated and highly specialized for their respective roles. In primitively eusocial Hymenoptera, morphological differentiation of the castes is weaker and many differences between queens and workers are behavioural^1^. As a relatively primitively eusocial species, the bumblebee *Bombus terrestris* occupies an intermediate position with respect to caste differences, in that queens and workers are similar in external morphology and ovariole number but differ in body mass, physiology and behaviour, with workers being unable to mate^24^,^25^. The genus *Bombus* shares a primitively eusocial common ancestor with *Apis*^26^. The extant clades descended from this common ancestor are either advanced eusocial (Apini, comprising *Apis*; and Meliponini), primitively eusocial (Bombini, comprising *Bombus*) or non-eusocial (Euglossini)^26^. Hence *Bombus* represents the closest extant model for the primitively eusocial common ancestor with *Apis*, albeit one that, since the split, will have undergone independent evolution towards its own form of eusociality. For these reasons, *B. terrestris* makes an excellent system in which to investigate the molecular basis of queen-worker caste differentiation and determination, including the role of miRNAs, in the evolutionary trajectory between primitive and advanced eusociality.

In *B. terrestris*, queens and workers develop through four larval instars, identifiable by mass and head width^27^. Caste fate (adult phenotype) is determined, i.e. differentiation is initiated, early in the development of female larvae^24^,^28^. Larvae are irreversibly determined from a totipotent state (i.e. in which larvae are capable of developing into either caste) towards worker development provided that, during a sensitive larval phase for 3–5 days after hatching (in the second instar), the colony queen is present and emitting a pheromonal signal^27^,^29^,^30^,^31^. It is hypothesized that, later in the colony cycle, the queen stops producing the pheromone, so allowing female larvae to develop as queens^30^,^31^,^32^. Two key hormone groups involved in this process are juvenile hormone (JH) and the ecdysteroids, with high larval levels of each hormone being associated with queen development^27^,^28^,^33^,^34^. Queen-destined larvae undergo JH peaks in their second and third instars that delay larval moult, so extending their developmental time and leading to the greater adult body size of queens relative to workers^27^,^28^. However, some female larvae developing in the absence of queen pheromone may still become worker-like adults if, during the fourth instar, they receive insufficient food^35^. By contrast, worker-destined larvae lose totipotency and cannot change to the queen pathway once they have passed the second instar without a JH peak, i.e. if they have passed through the sensitive phase in the queen’s presence^28^.

Overall, this system of caste determination differs from that of *A. mellifera*, since, in *B. terrestris*, the queen’s presence (via queen pheromone) and amount of food jointly influence caste, whereas in *A. mellifera* totipotent female larvae must be fed specialized food (royal jelly) to develop as queens^36^. However, the extent to which the molecular basis of caste determination differs across the two taxa remains unclear. By profiling early- and late-instar queen- and worker-destined female larvae for miRNAs in *B. terrestris*, we were able, for the first time, to investigate the role of miRNAs in queen-worker caste determination in a relatively primitively eusocial species, to characterize miRNA profiles in larval development before and after the change from totipotency to fixity in caste fate, and to compare the roles of miRNAs across the gradient of eusociality represented by *B. terrestris* and *A. mellifera*.

## Results

### SRNA-seq reveals caste-associated miRNAs in *B. terrestris*

**Figure 1:**
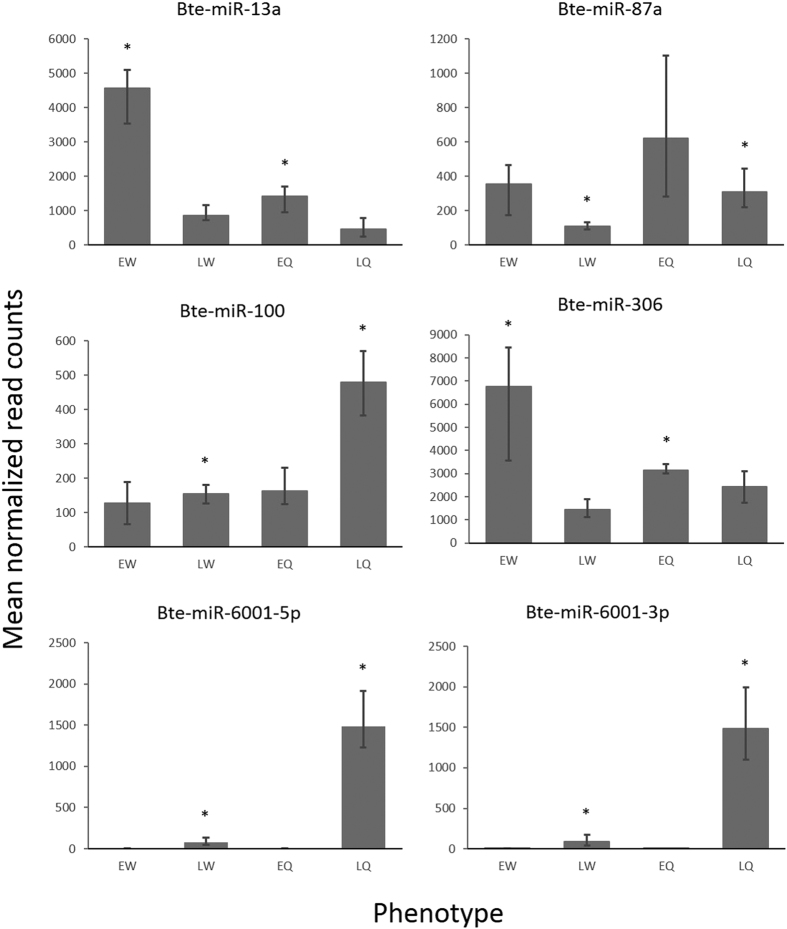
Mean normalized read counts for six miRNAs obtained by sRNA-seq of pooled whole body preparations of female larvae of *Bombus terrestris* and identified as being differentially expressed between queen- and worker-destined larvae. Error bars represent the range of the normalized read counts across three replicate libraries for each phenotype (all libraries excluding EW4, LW4, EQ2 and LQ2; see Supplementary Information). Phenotype: EW, early-instar worker-destined larvae; LW, late-instar worker-destined larvae; EQ, early-instar queen-destined larvae; LQ, late-instar queen-destined larvae. Asterisks indicate, within instars between caste phenotypes, pairs of phenotypes showing differential expression (i.e. when log_2_(OFC) ≥ 1).

We used deep sequencing of small RNAs (sRNA-seq) from queen- and worker-destined larvae that were collected from their colonies either while still totipotent (early-instar larvae) or after their caste fate was fixed (late-instar larvae). Each phenotype was sequenced in four biological replicates, comprising paired but separate pools of early- and late-instar larvae within each caste pathway from each of eight separate colonies, yielding 16 samples in total. We previously used worker-destined larvae of both instars to characterize 101 miRNAs in *B. terrestris* female larvae, of which 16 were new miRNAs (i.e. not isolated in any other organism) and 85 were shared with *A. mellifera* and so appeared conserved across the corbiculate bees^37^. In the present study, sRNA-seq of queen- and worker-destined *B. terrestris* female larvae returned six conserved miRNAs that had high normalized read counts (≥200 reads per 4 million reads) and that were differentially expressed between the caste phenotypes. These were Bte-miR-13a, Bte-miR-87a, Bte-miR-100, Bte-miR-306, Bte-miR-6001–5p and Bte-miR-6001-3p (Fig. 1; Supplementary Information), with the last two miRNAs being, respectively, the 5′ and 3′ arms of the Bte-miR-6001 duplex. Bte-miR-13a and Bte-miR-306 were most highly expressed in worker-destined larvae in early but not late larval instars (Fig. 1). Bte-miR-87a, Bte-miR-100, Bte-miR-6001-5p and Bte-miR-6001-3p were most highly expressed in queen-destined larvae in late but not early larval instars (Fig. 1). Both Bte-miR-6001-5p and Bte-miR-6001-3p showed the highest degree of differential expression (16.5 and 15.8 fold change between caste phenotypes, respectively), while the other four miRNAs showed only 2.2–3.2 fold change between expression levels between caste phenotypes (Fig. 1).

### Northern blot validation of caste-associated miRNAs

**Figure 2:**
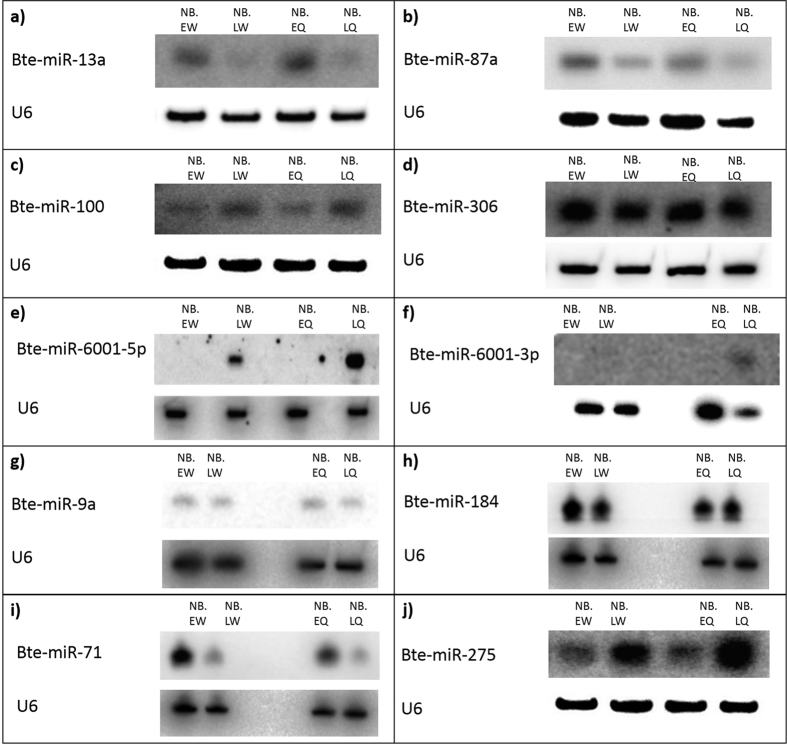
Northern blot expression profiles for ten miRNAs, and a control probe (U6), in pooled whole body preparations of female larvae of *Bombus terrestris*. EW, early-instar worker-destined larvae; LW, late-instar worker-destined larvae; EQ, early-instar queen-destined larvae; LQ, late-instar queen-destined larvae. Prefix ‘NB’ denotes a sample for Northern blot (Supplementary Table S3).

We used Northern blots to validate the six miRNAs found to be differentially expressed between caste phenotypes on the basis of the sRNA-seq data. We also used Northern blots to validate four other miRNAs selected on the basis of homology to miRNAs associated with larval or pupal caste differences in *A. mellifera*^20^,^21^,^22^,^23^ (Supplementary Table S1) or differential expression between larval instars within castes in *B. terrestris* (Supplementary Information). Each phenotype was analysed in at least two of five biological replicates drawn from paired but separate pools of early- and late-instar larvae within each caste pathway from each of ten separate colonies. The patterns of expression of Bte-miR-6001-5p and Bte-miR-6001-3p observed in the sRNA-seq were confirmed by the Northern blots. Specifically, both miRNAs were more highly expressed in late-instar queen- versus worker-destined larvae (Fig. 2). By contrast, the Northern blots failed to validate the four remaining *B. terrestris* miRNAs that had been identified as differentially expressed between caste phenotypes by sRNA-seq (Fig. 2), i.e., while the miRNAs were expressed at a level detectable by Northern blot, the assay showed no evidence of their being differentially expressed between the caste phenotypes (Fig. 2). In addition, the Northern blots similarly failed to validate the four miRNAs associated with larval or pupal caste differences in *A. mellifera* or with differences between larval instars within castes in *B. terrestris* (Fig. 2).

**Figure 3:**
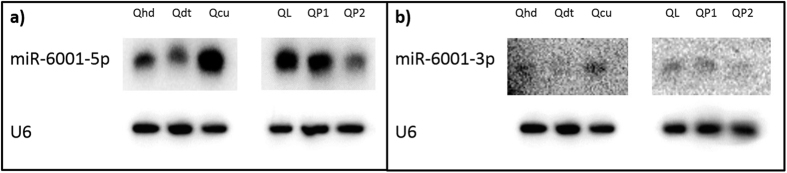
Northern blot expression profiles for two miRNAs, (**a**) miR-6001-5p and (**b**) miR-6001-3p, and a control probe (U6), in *Bombus terrestris* late-instar queen-destined larvae and queen-destined pupae. The miR-6001-3p probe produced a weaker signal than the miR-6001-5p probe; however, both probes showed the same pattern of gene expression. Qhd, queen-destined larva, head; Qdt, queen-destined larva, digestive tract; Qcu, queen-destined larva, cuticle; QL, queen-destined larva, whole body preparation; QP1, early queen pupa, whole body preparation; QP2, late queen pupa, whole body preparation.

We also used Northern blots to investigate the expression patterns of Bte-miR-6001-5p and Bte-miR-6001-3p in specific tissues of late-instar queen-destined larvae (head, digestive tract and outer cuticle) and in whole bodies of queen-destined larvae and pupae. We found that, in late-instar queen-destined larvae, both miRNAs were most highly expressed in outer cuticle (Fig. 3). In addition, both miRNAs were expressed at lower levels in late queen pupae compared to late-instar queen-destined larvae and early queen pupae (Fig. 3).

### Genomic context of caste-associated miRNAs

**Figure 4:**
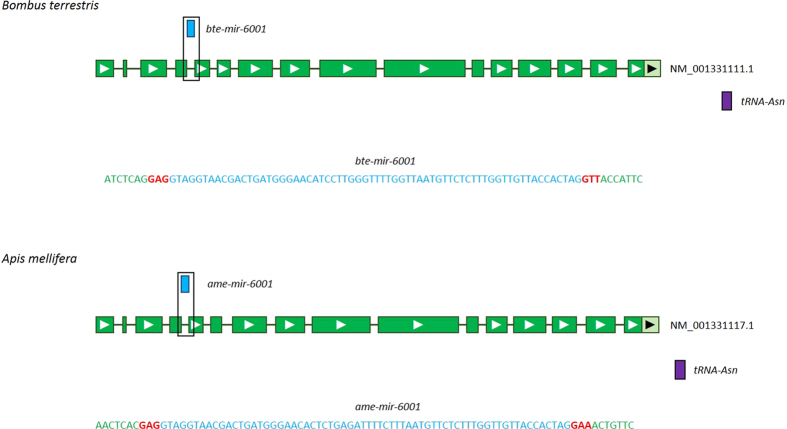
Schematic diagram of very high density lipoprotein (*Vhdl*) and mir-6001 in *Bombus terrestris* and *Apis mellifera*. Light blue boxes represent mir-6001, green boxes represent the predicted exons of *Vhdl*, dark green lines represent the introns, and the purple box the neighbouring tRNA sequence in both genomes. The black box around mir-6001 and the fourth intron of *Vhdl* represents the sequence shown below each gene schematic. In these sequences, green characters represent nucleotides at the end of the fourth and start of the fifth exons of *Vhdl*; red characters, the end of the intron-exon splice junctions; and blue characters, the predicted mir-6001 precursor sequence containing the miR-6001 miRNA duplex. Gene model in schematic diagram modified from the NCBI database; RefSeq accession number (on the right of the schematics) from the NCBI database.

Genome scanning for the gene *bte-mir-6001* against the *B. terrestris* genome^37^ showed that this miRNA and its precursor sequence comprise the entire fourth intron of *very high density lipoprotein (Vhdl*, RefSeq accession number: NM_001331111.1; Fig. 4), which is a gene with homology to *Vitellogenin*. This signifies that this miRNA is a mirtron, i.e. an intronic miRNA that replaces the Drosha cleavage with splicing to generate the pre-miRNA^38^,^39^. A BLAST search of this sequence against the *A. mellifera* genome^40^ revealed that the mirtron was conserved in the same intron of the same gene in *A. mellifera* (Fig. 4).

### Target prediction of caste-associated miRNAs

Target prediction for Bte-miR-6001-5p and Bte-miR-6001-3p showed that many putative targets for these miRNAs are genes involved in development and reproductive differentiation in *Apis* and *Drosophila*, including genes associated with ovary and oocyte development, neurodevelopment, larval development and larval moulting (Table 1). Specifically, predicted targets of Bte-miR-6001-5p included *ecdysone-induced protein 75* and *ferredoxin* (Table 1).

## Discussion

We have isolated and validated two miRNAs (Bte-miR-6001-5p and Bte-miR-6001-3p) that are more highly expressed in queen- than in worker-destined late-instar larvae in *B. terrestris* (Figs 1and 2). This expression pattern suggests that these miRNAs are associated with queen-worker caste determination and/or differentiation in larvae. Such a role is consistent with expression differences in these miRNAs occurring in late- but not early-instar larvae (Figs 1and 2), i.e. after the sensitive phase of caste determination in the second instar in which worker-destined larvae lose totipotency^28^, and with the decline in their expression in late queen pupae (Fig. 3). It is also consistent with higher expression of Bte-miR-6001-5p and Bte-miR-6001-3p in the cuticle of late-instar queen-destined larvae (Fig. 3), given that differentiation of queens in *B. terrestris* involves delayed moulting of later larval instars^28^ and that, in the *B. terrestris* transcriptome, genes involved in larval cuticular biogenesis show elevated expression relative to other genes^41^. In addition, a role in caste determination for Bte-miR-6001-5p and Bte-miR-6001-3p matches the finding that the predicted targets of Bte-miR-6001-5p include *ecdysone-induced protein 75* and *ferredoxin*, both of which have been associated with responses to ecdysone in *Drosophila*^42^,^43^. Ecdysone is an ecdysteroid, a well-known family of insect hormones that mediate many processes including caste determination in both *A. mellifera*^44^ and *B. terrestris*^33^.

A role for miRNAs in caste determination in *B. terrestris* and other eusocial Hymenoptera is consistent with miRNAs having been found to mediate the development of alternative adult phenotypes in other insects^18^,^19^, although miRNAs may not underpin behavioural caste differences in adults in other primitively eusocial Hymenoptera^45^. In *B. terrestris*, one possibility is that one or both arms of the Bte-miR-6001 duplex targets a protein-coding gene that prevents larvae from developing along the queen-destined pathway, so that, by silencing the gene, the miRNA allows larvae to develop as queens. The detailed mechanism by which this occurs remains open for future investigation, but could involve a peak in Bte-miR-6001 responding to the JH peaks exhibited by queen-destined larvae, or to the absence of queen pheromone, or the greater amount of food received by late-instar queen-destined larvae^27^,^28^,^29^.

Our finding that *bte-mir-6001* is a mirtron within *Vhdl* suggests a novel link between miRNA regulation of caste determination and the *Vitellogenin* family of genes, including the gene *Vitellogenin (Vg*), to which *Vhdl* has sequence homology. Vitellogenins are an important class of nutritive proteins induced by JH and linked to reproduction in numerous insects^46^, including eusocial Hymenoptera. For example, in bees, vitellogenins are associated with ovarian activation in adult female *Bombus*^47^,^48^ and *Apis*^49^ and with larval queen-worker caste differentiation in *Apis*^9^. These associations suggest that *Vhdl* is also a candidate for a caste-associated gene in eusocial Hymenoptera in general.

Despite validating the sequencing results for the Bte-miR-6001 duplex, the Northern blots failed to confirm expression patterns for four other *B. terrestris* miRNAs that were differentially expressed according to sRNA-seq (Bte-miR-13a, Bte-miR-87a, Bte-miR-100, Bte-miR-306; Fig. 2). This could have occurred for several reasons. First, the sequencing results could have been false positives, with the Northern blot results reflecting a true lack of differential expression between the caste phenotypes. Second, it is possible that differential expression was present but that the low absolute levels of expression of these miRNAs (in contrast to those of the miR-6001 duplex) meant that differential expression was not detected by the Northern blots. Third, a high level of variation between replicates in the absolute level of expression of these four miRNAs could have masked any differential expression present. Previous studies^50^,^51^, including a study on miRNAs differentially expressed between *Apis* behavioural phenotypes^51^, have also found that Northern blots do not always show the same results as sRNA-seq analysis.

Our results show that the miRNAs associated with caste differentiation in *Bombus* and *Apis* exhibit little overlap. Specifically, the Northern blots showed that the homologues of four miRNAs associated with queen-worker caste determination in *A. mellifera* (Ame-miR-9a, -184, -71 and -275; Supplementary Table S1) were not differentially expressed between queen- and worker-destined larvae in *B. terrestris* (Fig. 2). Other studies have shown that, in *A. mellifera*, Ame-miR-6001-5p was not differentially expressed between queen- and worker-destined larvae^21^ and that Ame-miR-6001-3p was weakly differentially expressed between castes in larvae^21^ or was differentially expressed between female and male larvae but not between castes in larvae^22^. In sum, four out of four caste-associated miRNAs in *A. mellifera* that we investigated in *B. terrestris* lack such a role in *B. terrestris*, and at least one of two caste-associated miRNAs in *B. terrestris* lack this role in *A. mellifera*. Moreover, our previous profiling of miRNAs in *B. terrestris* showed that there was little overlap in the total set of miRNAs in the genomes of the two species, with *A. mellifera* having up to 103 miRNAs in its genome that are not found in the genome of *B. terrestris*^37^. Overall, therefore, our findings suggest that, within the bees, miRNAs are associated with caste determination and differentiation relatively early in eusocial evolution but that the role of individual miRNAs is not generally conserved as eusocial evolution proceeds. Hence, as well as the emergence of novel (taxonomically restricted) genes underpinning eusocial evolution across the Hymenoptera^45^,^52^,^53^, novelty of function could be a feature of the molecular basis of eusocial evolution. Further investigations, as well as focusing on the mechanism by which the Bte-miR-6001 duplex might affect caste, on its putative targets and on *Vhdl*, would therefore benefit from considering how its role has been modified during the change from primitive to advanced eusociality in bees.

## Methods

### Sample Collection

We collected female larvae from *B. terrestris* colonies between May 2011 and December 2013 (see Supplementary Information for full details of all methods). We removed the queen from a sample of colonies to generate queen-destined larvae and we obtained worker-destined larvae from the remaining, queenright (with a queen) colonies. Caste fate of all sampled larvae was verified by tracking the caste fate of larvae retained in the same batch and allowed to develop to adulthood^8^. We thereby collected larvae with four different phenotypes: early- and late-instar worker-destined larvae and early- and late-instar queen-destined larvae. Colonies were separated into three cohorts: cohort 1 (8 colonies; Supplementary Table S2) was used to produce RNA for sRNA-seq; cohort 2 (10 colonies; Supplementary Table S3) was used to produce RNA for Northern blot validation; and cohort 3 (1 colony) was used to produce larval tissues (head, cuticle, digestive tract) and pupae (early-pupae and late pupae) for Northern blot investigation of miR-6001-5p and miR-6001-3p in queen-destined developmental pathways.

We pooled larvae that had the same phenotype and that were collected from the same colony within the total set of colonies, and then extracted total RNA from each pool using Trizol. Within each caste pathway, early- and late-instar larvae were sampled from the same colony; therefore instar stages were paired within colonies and each colony pool was a biological replicate for each phenotype. In this way, we created four biological replicates for each phenotype from cohort 1 for sRNA-seq (Supplementary Tables S2, S4) and five biological replicates from cohort 2 for Northern blots (Supplementary Tables S3, S5). We used RNA from the four biological replicates of the four phenotypes from cohort 1 to generate 16 cDNA libraries (Supplementary Table S4). We prepared the libraries using the TruSeq small RNA library preparation kit v.1.5 (Epicentre Technologies, Madison, Wisconsin, USA) with HD modifications to the 3′ adapter to reduce sequencing bias^54^. The libraries were sequenced using Illumina sequencing on the HiSeq2000 platform.

### Bioinformatic Analysis

The 16 sRNA-seq libraries produced a total of 86 million reads (2.4–6.3 million reads per library; Supplementary Table S4). After excluding reads that contained unassigned nucleotides, we trimmed the 3′ adapter sequence and the HD signature from each read. We excluded sequences shorter than 16 nucleotides from further analysis. We mapped remaining reads to the *B. terrestris* genome v.1.0^37^, full length, allowing one mis-match and no gaps, using the software *PatMaN*^55^. On average, 69.4% of the redundant reads (all reads) and 54.9% of the non-redundant reads (unique reads) mapped to the *B. t. terrestris* genome across all 16 libraries (Supplementary Table S4). As expected, a high average proportion of these genome-matching reads was incident to miRNAs (24.7% of the redundant reads, Supplementary Table S6). We normalized the 16 libraries using the read count per total normalization method^56^,^57^,^58^, with the normalization total for each library set at 4 million, which was the median total read count (rounded up to the nearest million) of the accepted reads across all libraries^57^. After checking the efficiency of the normalization, we excluded four libraries (one from each phenotype) from further analysis (see Supplementary Information). We aligned (full length, allowing up to two mis-matches and no gaps) the sequences to the Hexapoda miRNAs listed on miRBase v.21^59^. Following^60^,^61^, we identified the differentially expressed miRNAs using a maximal expression interval approach, and the degree of differential expression was determined using the log offset fold change (OFC) method across all libraries for pairwise comparisons of phenotypes. To isolate miRNAs associated with caste, we compared worker- and queen-destined larvae within each instar. To isolate miRNAs associated with development, we compared early- and late-instar larvae within each caste pathway. We defined miRNAs as differentially expressed when log_2_(OFC) ≥ 1 between phenotypes^60^.

We used miRanda^62^ to identify a list of potential targets of the two miRNAs that had a validated pattern of differential expression (Bte-miR-6001-5p, Bte-miR-6001-3p). We identified the putative function of each target using the descriptions presented in NCBI (http://www.ncbi.nlm.nih.gov/) and FlyBase (http://flybase.org/).

### Northern Blots

To validate the expression of miRNAs that were identified as differentially expressed between castes in cohort 1, we used Northern blots to probe for miRNAs in RNA extracted from pooled samples from cohort 2 (Supplementary Tables S5, S7). For each miRNA, we produced Northern blots comparing the expression in each phenotype in at least two biological replicates. The miRNA expression was considered validated if both biological replicates showed a pattern of differential expression that was the same as the sRNA-seq. In addition, we used Northern blots of RNA extracted from larval and pupal samples in cohort 3 to investigate the tissue- and stage-specificity of the two caste-associated miRNAs that were validated by the Northern blots (Bte-miR-6001-5p and Bte-miR-6001-3p). For all Northern blots U6, a stably expressed nuclear RNA, was used as a loading control.

### Data Access

The raw sRNA-seq data and raw counts have been submitted to GEO NCBI (GEO accession numbers: GSE64512 and GSE77870).

## Additional Information

**How to cite this article:** Collins, D. H. *et al*. MicroRNAs Associated with Caste Determination and Differentiation in a Primitively Eusocial Insect. *Sci. Rep.* **7**, 45674; doi: 10.1038/srep45674 (2017).

**Publisher's note:** Springer Nature remains neutral with regard to jurisdictional claims in published maps and institutional affiliations.

## Supplementary Material

Supplementary Information

## Figures and Tables

**Table 1:** Possible target genes and their functions for two caste-associated miRNAs isolated from female larvae of *Bombus terrestris*.

MicroRNA	Gene name	Accession number	Target Gene Biological Function (Gene Ontology terms from FlyBase)
Bte-miR-6001-5p	*Ecdysone-induced protein 75 (E75)*	XM_003398942.2	Antimicrobial humoral response, Ecdysis, Molting cycle, Regulation of ecdysteroid metabolic process, regulation of gene expression, regulation glucose metabolic processes, response to ecdysone
	*Ephrin receptor tyrosine kinase (EphR), transcript variant X12*	XM_012315751.1	Melanotic encapsulation of foreign target, Mushroom body development, Peripheral nervous system development, Regulation of glucose metabolic process
	*Vacuolar protein sorting 13B (Vps13B)-like*	XM_012317130.1	Protein targeting to the vacuole
	*Lysine-specific demethylase (lid)*	XM_003393890.2	Chromatin organization, Gene silencing, Heterochromatin organization, Histone H3-K4 demethylation, Imaginal disc-derived wing vein specification, Oogenesis, Positive regulation of methylation-dependent chromatin silencing, Regulation of Notch signaling pathway
	*Ferredoxin (fdxh)*	XM_003393566.2	Positive regulation of ecdysteroid biosynthetic processes, Pupariation
	*Parvulin prolyl isomerase 1 (parv 1)*	XM_003397622.2	Epidermal growth factor receptor (Egfr) pathway
	*Roughened (R)*	XM_003397072.2	Adherens junction assembly, Cell adhesion, Dorsal closure, Establishment of ommatidial planar polarity, Germ-line stem cell population maintenance, Hemocyte migration, Positive regulation of cell-cell adhesion, Rap protein signal transduction, Regulation of cell shape, Regulation of embryonic cell shape, Substrate-dependent cell migration, Cell extension
	*RNA-dependent helicase p7*	XM_003397280.2	RNA helicase
	*Translationally controlled tumor protein (Tctp)*	XM_003397970.2	Cellular response to gamma radiation, Double-strand break repair, Intra-S DNA damage checkpoint, Mitotic G2 DNA damage checkpoint, Positive regulation of cell size, Positive regulation of histone phosphorylation, Positive regulation of multicellular organism growth
	*No extended memory (nemy)*	XM_012312185.1	Imaginal disc-derived wing morphogenesis, Locomotory behaviour, Memory
Bte-miR-6001-3p	*Distal-less (DII)*	XM_012315363.1	Antennal development, determination of ventral identity, imaginal disc-derived appendage morphogenesis, imaginal disc-derived leg morphogenesis, imaginal disc-derived wing margin morphogenesis, leg disc proximal/distal pattern formation, mushroom body development, negative regulation of gene expression, olfactory behaviour, factory nerve development, positive regulation of transcription, DNA-templated, proboscis development, specification of organ identity
	*Gish casein kinase gilgamesh*	XM_003393759.2	Glial cell migration, negative regulation of actin nucleation, olfactory learning, ommatidial rotation, protein phosphorylation, regulation of endocytic recycling, regulation of establishment of planar polarity, response to mechanical stimulus, sensory perception of pain, sperm individualization, spermatogenesis, Wnt signaling pathway
	*beta4GalNAcTA beta 1,4-N-acetylgalactosaminyltransferase A*	XM_003402833.2	Adult locomotory behaviour, glycolipid biosynthetic process, glycosphingolipid biosynthetic process, N-acetylglucosamine metabolic process, neuromuscular junction development, sperm individualization
	*Baboon (babo)*	XM_012318461.1	Activin receptor signaling pathway, axon guidance, determination of adult lifespan, eye-antennal disc morphogenesis, imaginal disc-derived wing morphogenesis, mushroom body development, negative regulation of autophagy, neuroblast proliferation, neuron development, positive regulation of BMP signaling pathway,positive regulation of pathway-restricted SMAD protein phosphorylation, regulation of glucose metabolic process, regulation of mitotic cell, response to UV
	*RNA-dependent helicase p72*	XM_003397280.2	RNA helicase

Gene ontologies from FlyBase and gene annotations and RefSeq accession numbers from the NCBI database.
