# Supplementary material for: MicroRNAs Associated with Caste Determination and Differentiation in a Primitively Eusocial Insect
Source: Sci Rep. 2017 Mar 31;7:45674. doi: 10.1038/srep45674 (PMC5374498; doi:10.1038/srep45674)
Supplement: Supplementary Information [file srep45674-s1.pdf]

MicroRNAs Associated With Caste Determination and Differentiation in a Primitively Eusocial Insect

Supplementary Information

David H. Collins[1]*, Irina Mohorianu[1, 2], Matthew Beckers[2], Vincent Moulton[2], Tamas Dalmay[1], and Andrew F. G. Bourke[1]

[1]School of Biological Sciences, University of East Anglia, Norwich Research Park, Norwich NR4 7TJ, UK

[2]School of Computing Sciences, University of East Anglia, Norwich Research Park, Norwich NR4 7TJ, UK

*Correspondence: David.Collins@uea.ac.uk

Supplementary Methods

Sampling of queen- and worker-destined larvae

We obtained 63 *Bombus terrestris* colonies in three cohorts between May 2011 and December 2013. Cohort 1 consisted of 40 colonies obtained from Syngenta Bioline B.V. (Weert, The Netherlands) in May 2011 and was used to generate queen- and worker-destined larvae for deep sequencing of small RNAs (sRNA-seq) (Table S2). MicroRNAs (miRNAs) are the best characterised class of sRNAs, and occupy the majority of the sequencing space in sRNA-seq libraries. Therefore we focussed our analysis on this class of sRNA. Cohort 2 consisted of 20 colonies obtained from the same supplier in April 2012 and was used to generate queen- and worker-destined larvae for miRNA validation by Northern blot (Table S3). Cohort 3 consisted of 3 colonies obtained from Koppert B.V. (Berkel en Rodenrijis, The Netherlands) in December 2013 and was used to generate RNA from specific larval tissues and from queen pupae to identify the stage- and tissue-specificity of caste-differentiated miRNAs. Colonies were reared in plastic boxes (measuring 20 cm × 20 cm × 15 cm) kept in standard conditions (28°C, 60% R.H., under red light)[1] and supplied *ad libitum* with sugar-syrup (Syngenta Bioline B.V.) and freeze-dried pollen (Koppert B.V.).

On arrival, each colony in all three cohorts contained a single queen that had not yet produced any workers. This allowed us to record the date of first worker eclosion (when workers first emerge from their pupae), which in turn permitted us to estimate the date of the 'switch point'. In the colony cycle of *B. terrestris*, the switch point is defined as the point when the queen starts to produce male eggs; it usually occurs 2-4 weeks after the date of first worker eclosion[1,2]. In the present study it was necessary to estimate the switch point to ensure that only female larvae were sampled, given that male larvae are not easily distinguishable from worker-destined larvae and larvae present in post-switch point colonies could have been male. Accordingly, our strategy for sampling queen- and worker-destined larvae involved sampling early-instar larvae only from pre-switch-point colonies (i.e. from colonies up to 14 days after first worker eclosion) and late-instar larvae only from larval batches that had been early-instar larvae during this pre-switch period. With this condition, our strategy involved the following steps: (a) sampling (by removal) approximately 50% of each batch of early-instar larvae produced per colony, and allowing the remaining 50% to develop into late-instar larvae; (b) of these late-instar larvae, sampling approximately 50% per colony, and leaving the remaining 50% to develop into adults. We then monitored the development of the unsampled late-instar larvae until their eclosion. This final step allowed us to verify the caste fate (adult phenotype) of sampled larvae by recording the caste fate of unsampled larvae within the same batches. We also individually weighed all sampled larvae on a balance (A&D, Abingdon, UK) to confirm the instar of all larvae and the caste of late-instar larvae.

Cohort 1: samples for sRNA-seq

For cohort 1, we randomly divided the colonies into two groups of 20, with one group being reared to produce worker-destined larvae and the other group being reared (by queen removal) to produce queen-destined larvae. Once the first workers had eclosed, we photographed each colony daily at 17:00 until the first males eclosed (28-93 days after receiving the colonies, except in the case of one colony, which was excluded, in which adult males eclosed at the same time as the first workers eclosed). Using the photographs, we determined the locations of the developing brood and any newly-laid eggs, allowing us to follow the development of individual larvae after the first instar in each of the experimental colonies. In total, seven colonies were excluded from the experiment (five in which the queen died one or two days after the experiment began, one that contained workers on arrival and

the colony in which adult males eclosed at the same time as the first workers eclosed), leaving 33 colonies in cohort 1.

We retained the colony queen in 13 of the 33 colonies (henceforth queenright colonies, generating worker-destined larvae) and removed the colony queen from the remaining 20 colonies (henceforth queenless colonies, generating queen-destined larvae). Queen removal was carried out 14 days after first worker eclosion. Because *B. terrestris* queens produce a queen pheromone in the presence of which female larvae develop into workers, queen removal induces queen production[3-5]. In the queenright colonies, we removed up to half of the 1st or 2nd instar larvae (1-3 days old) every 2-3 days until 10-14 days after first worker eclosion, after which further early-instar larvae were not sampled as colonies were likely to have reached their switch point. In the queenless colonies, we removed up to half of the 1st or 2nd instar larvae every 2-3 days for 6 days after the queen was removed. Because *B. terrestris* eggs hatch 5 days after they are laid[6], any eggs present after this time would have been male eggs produced by egg-laying workers, and so sampling of early-instar larvae was then halted. In both queenright and queenless colonies, we allowed all unsampled larvae to develop to the 4th (final) instar, which is beyond the point in larval development when caste fate has been irreversibly determined[7]. We then sampled approximately half of the 4th instar larvae from both sets of colonies.

Among the 13 queenright colonies, eight colonies occurred in which all of the unsampled larvae eclosed as workers. These eight colonies were maintained for a further month after all focal larvae had pupated and eclosed as workers, which allowed us to determine that no queens or males eclosed for two weeks or more after final larval sampling. This meant that all sampled larvae in these colonies would have been very likely to develop into workers. We selected as colonies providing worker-destined larvae the four of these colonies that had the highest sample sizes of early-instar and late-instar larvae. In the four selected colonies, 100% (148/148) of unsampled late-instar larvae eclosed as workers (Table S2). All 1st and 2nd instar larvae sampled from these colonies were classified as early-instar worker-destined larvae and all 4th instar larvae were classified as late-instar worker-destined larvae. The masses (mean (range) mg, n = number of larvae) of the sampled worker-destined larvae were as follows: early-instar larvae: 7.5 (3.5–15.3) mg, n = 164; late-instar larvae: 252.4 (107.0–454.4) mg, n = 85. These values confirmed that the early-instar larvae were 1st or early 2nd instar larvae, as 2nd instar *B. terrestris* worker-destined larvae have been shown to have a maximum mass of 39.5 mg[3,4]. Likewise, these values confirmed that the late-instar larvae were 4th instar larvae, as *B. terrestris* worker-destined 4th instar larvae have been shown to have a minimum mass of 95.8 mg[3,4].

Among the 20 queenless colonies, eight colonies produced queens. We sampled queen-destined larvae from the four colonies among these eight that produced the highest percentages of adult queens from the unsampled late-instar larvae. In three colonies, this percentage was 100% (38/38 larvae; Table S2). In the fourth colony (QL-14), it was 85% (11/13 larvae; Table S2), i.e. two unsampled larvae eclosed as workers. However, retrospective calculation of the proportion of all late-instar larvae (sampled and unsampled) that were queen-destined in this colony showed this proportion to be 95% (38/40; Table S2). All sampled late-instar larvae from all four colonies could be identified as queen-destined, because in *B. terrestris* it is possible to tell late-instar worker- and queen-destined larvae apart by mass alone, with queen-destined larvae having a mass approximately four times greater [3]. The masses (mean (range) mg, n = number of larvae) of queen-destined larvae sampled from the four colonies were as follows: early-instar larvae: 9.3 (7.2–14.7) mg, n =

76; late-instar larvae: 1168.7 (772.5–1455.4) mg, n = 65. These values confirmed that the early-instar larvae were 1st or early 2nd instar larvae, as 2nd instar *B. terrestris* queen-destined larvae have been shown to have a maximum mass of 64.0 mg[3,4]. Likewise, these values confirmed that the late-instar larvae were 4th instar larvae, as *B. terrestris* queen-destined 4th instar larvae have been shown to have a minimum mass of 256.2 mg[3,4].

In summary, in the four selected queenright colonies, we monitored 397 larvae in total. Of these we sampled 164 early-instar worker-destined larvae and 85 late-instar worker-destined larvae. Of the remaining 148 unsampled larvae reaching the late instar, 100% eclosed as workers (Table S2). In the four selected queenless colonies, we monitored 192 larvae in total. Of these we sampled 76 early-instar queen-destined larvae and 65 late-instar queen-destined larvae. Of the remaining 51 unsampled larvae reaching the late instar, 96% eclosed as queens, i.e. all except for two larvae eclosing as workers (Table S2). These data confirmed that larvae sampled from each colony type would have developed along the expected caste trajectory in the vast majority of cases.

Within each colony, instar and caste, we pooled sampled larvae as whole bodies and then snap-froze them in liquid nitrogen and crushed them into a mass of fine frozen particles using a chilled ceramic mortar and pestle. We dissolved the pooled mixture into Trizol reagent (Invitrogen, Carlsbad, California, USA), using 1.5 ml of Trizol for every 100 mg of tissue. We snap-froze the resulting mixtures in liquid nitrogen and stored them at -80°C. We pooled larvae because sRNA-seq required up to 2 µg of total RNA per phenotype, which was more RNA than the early-instar larvae yielded individually. Pooling also meant that the miRNA read counts (the number of sequences returned from the sRNA-seq) of different samples reflected differences between phenotypes rather than individuals[8]. To ensure that the samples were biological replicates representing colony variation, each sample was created by pooling larvae from the same colony and phenotype. The samples consisted of 10–44 early-instar larvae per colony and 5–27 late-instar larvae per colony. These procedures generated 16 samples, representing four biological replicates (colonies) per each of the four phenotypes, with each colony providing one early-instar and one late-instar sample for each caste fate (Tables S3, S5).

Cohort 2: samples for Northern blots

Colonies in cohort 2 were reared and sampled following the same procedures as those used for cohort 1. The queen died soon after arrival in two colonies, which were therefore excluded from the experiment. Of the remaining 18 colonies, we maintained five in a queenright condition (to produce worker-destined larvae) and removed the colony queen from the remaining 13 colonies (to produce queen-destined larvae). We isolated worker- and queen-destined larvae following the methods used for cohort 1. In the five queenright colonies (Table S3), we monitored 551 larvae in total. Of these we sampled 223 early-instar worker-destined larvae and 92 late-instar worker-destined larvae. Of the remaining 236 unsampled larvae reaching the late instar, 235 (99.6%) eclosed as workers (a single male eclosed from these larvae in one colony; Table S3). In eight of the 13 queenless colonies, large numbers of workers eclosed and these colonies were therefore excluded from experiment. In the five remaining queenless colonies (Table S3), we monitored 284 larvae in total. Of these we sampled 122 early-instar queen-destined larvae and 56 late-instar queen-destined larvae. Of the remaining 106 unsampled larvae reaching the late instar, 103 (97.1%) eclosed as queens (a single worker eclosed from these larvae in each of three colonies; Table S3). As for cohort 1, because almost all unsampled larvae from each selected colony developed into the expected phenotype, we treated all sampled larvae from queenright

colonies as worker-destined and all sampled larvae from queenless colonies as queen-destined.

Larvae sampled from the cohort 2 colonies were pooled, snap-frozen and stored following the methods used for the cohort 1 larvae. Pooling was again conducted within colonies and phenotypes and was required because each Northern blot required up to 10 µg of total RNA. Samples consisted of 11–59 early-instar larvae per colony and 6–25 late-instar larvae per colony. This generated 20 samples, representing five biological replicates (colonies) per each of the four phenotypes, with each colony providing one early-instar and one late-instar sample for each caste fate (Tables S4, S6).

Cohort 3: samples for identifying miRNA stage- and tissue-specificity
Colonies in cohort 3 were reared following the methods used for the colonies yielding queen-destined larvae in cohorts 1 and 2. Following queen removal, one of the cohort 3 colonies produced 10 late-instar queen-destined larvae. We dissected four of the larvae into head, digestive tract and outer cuticle using a scalpel on a Perspex dissection board. The tissues were immediately separated from one another and homogenized in Trizol using a chilled ceramic mortar and pestle, frozen in liquid nitrogen, and stored individually at -80°C. We homogenized two of the larvae as whole-body preparations in Trizol, then froze them in liquid nitrogen and stored them individually at -80°C. The remaining four larvae were allowed to pupate in the colony. We sampled two of these two days after pupation ('early queen pupae') and the remaining two seven days after pupation ('late queen pupae'). We homogenized the four pupae in Trizol, froze them in liquid nitrogen, and stored them individually at -80°C. These procedures generated 18 individual samples for Northern blots: late-instar queen-destined larva head (4 samples); late-instar queen-destined larva digestive tract (4 samples); late-instar queen-destined larva outer cuticle (4 samples); late-instar queen-destined larva whole body preparation (2 samples); early queen pupa (2 samples); and late queen pupa (2 samples).

Molecular methods for sRNA-Seq
RNA extraction
We extracted total RNA from the larval-Trizol homogenates (separately for each sample) according to the instructions of the Trizol manufacturer (Invitrogen, Carlsbad, California, USA) with minor modifications. Following RNA extraction, we quantified total RNA using a Nanodrop 8000 spectrophotometer (ThermoFisher Scientific, Loughborough, UK). We measured the integrity of the RNA on a 1.2% agarose gel. All RNA used for sRNA-seq and Northern blots was pure (260/280 ratio > 1.6) and there was no evidence of degradation (data not shown).

cDNA library preparation
In cohort 1, we used total RNA extracted from the queen- and worker-destined larvae to construct 16 cDNA libraries (Tables S3, S5). The 16 libraries consisted of four libraries of early-instar worker-destined larvae (EW1–4), four of late-instar worker-destined larvae (LW1–4), four of early-instar queen-destined larvae (EQ1–4) and four of late-instar queen-destined larvae (LQ1–4) (Tables S3, S5). To make the cDNA libraries, we first enriched the total RNA for small RNAs (sRNA) (i.e. enriching the fraction of total RNA that was less than 200 bp in length) using a mirVana miRNA isolation kit (Ambion, Foster City, California, USA) according to the manufacturer's instructions. We then prepared the libraries using the

TruSeq small RNA library preparation kit v.1.5 (Epicentre Technologies, Madison, Wisconsin, USA) with modifications to the 3' adapter to reduce sequencing bias[9].

To ligate the adapters to the sRNA sequences, we followed the protocol provided with the TruSeq 1.5 library preparation kit with some modifications. Following preparation of the cDNA, we amplified each library with a unique index sequence using Illumina index primers (1-16). We tested the specificity of the PCRs by varying the cycle number for each library individually, choosing the cycle number that provided the clearest product when separated on an 8% polyacrylamide gel. We used the selected cycle number to prepare four PCR reactions for each library. We separated the PCR products on an 8% polyacrylamide gel, and then scanned the gel using a Molecular Imager FX Pro Plus (BIORAD, Hemel Hempsted, UK) and *Quantity One* software (BIORAD). We identified the 21-23mer miRNA band on the gel, and cut out the gel section that contained it. We then extracted the nucleic acids from the gel fragments and re-purified them using ethanol precipitation. To reduce the chances of adapter-adapter contamination, which is a common problem during library preparation[10], we separated the purified nucleic acid sample on a second 8% polyacrylamide gel and re-purified it by using ethanol precipitation and re-dissolving the pellet in ARG water. We took 1/6th of the purified product and separated it on a third gel to ensure that the miRNA band of interest was still present.

Sanger sequencing and sRNA-seq
To ensure that the cDNA libraries from the cohort 1 samples contained miRNAs, we cloned the construct from library EW-1 into a pGEMTeasy vector (Clontech, Saint-Germain-en-Laye, France) and then transformed it into DH5α super-competent *Escherichia coli* using blue-white colony staining. We then extracted the vectors containing the RNA fragment of interest by culturing the *E. coli* overnight, picking the white colonies and making mini-preps using the Qiagen mini-prep kit (Qiagen, Hilden, Germany) according to the manufacturer's instructions. We prepared ten samples using ready reactions (Life Technologies, Paisley, UK) and then sent them for Sanger chain-termination sequencing at the Earlham Institute (formerly The Genome Analysis Centre), Norwich Research Park, UK.

We used *FinchTV* sequence analysis software (Geospiza, Seattle, Washington) to identify each sequence. We were able to map six of the miRNA length sequences to the *B. terrestris* genome v.1.0[11]. One of the six sequences was identified as the miRNA Bte-miR-1, confirming that the libraries contained miRNAs (data not shown).

Finally, the 16 prepared cDNA libraries were shipped to BaseClear B.V (Leiden, The Netherlands), who conducted sRNA-seq of each library on the Illumina HiSeq2000 platform (single-ended reads, on 50 cycles). The sRNA-seq data are separated by each library and are available on the NCBI gene expression omnibus (GEO; https://www.ncbi.nlm.nih.gov/geo/). The worker-destined larvae data (GEO accession: GSE64512) were used to predict new miRNAs in the *B. terrestris* genome[11] and were made available following publication of the genomes, while the queen-destined larvae are available under the GEO accession number GSE77870. Each library is labelled on GEO according to its phenotype and replicate number, e.g. Early Queen Destined 1 corresponds to library EQ1.

Bioinformatic analysis
Quality checks and genome mapping
The sRNA-seq libraries from the cohort 1 samples returned 86 million reads across all 16 libraries (2.4–6.3 million reads per library). First, we filtered out all reads that contained

unassigned nucleotides; these represented less than 1% of the total number of reads in each sample (Table S4). Next, we trimmed the adapter sequences by matching the first 8 nt of the 5' end of the 3' adapter sequence; on average, the adapter was found for 80% of reads (Table S4). We then trimmed the 4 nt corresponding to the 3' HD adapter[9]; only sequences longer than 16 nt were kept for further analysis (on average 82.4% of the sequences with the adapter; Table S4). We term all reads remaining after these steps 'accepted reads'.

We also inspected the nucleotide composition of reads within the 21-24 nt range (Figure S1). No sequence motifs stood out for any size class; however, we observed that the 5' end of the 22mers and 23mers was enriched for thymine, which is the known hallmark of miRNAs (22mers) and their variants (23mers)[12]. In addition, there was no evidence of a single nucleotide dominating the sequences at any nucleotide position on the sequences (Figure S1).

Next we calculated the number of non-redundant reads (unique sequences) and the corresponding read complexities, defined as the ratio of the number of redundant reads (total number of reads produced from the sequencing) to non-redundant reads (Table S4).

We mapped all accepted reads to the *B. terrestris* genome v.1.0[11], full length, allowing one mis-match and no gaps, using *PatMaN*[13]. An average of 69.4% of the redundant reads and 54.9% of the non-redundant reads mapped to the genome across all 16 libraries (Table S4). The higher proportion of redundant reads mapping to the genome indicated that the majority of mapped reads were reads with high abundance rather than 'noise', and therefore that the mapped reads were likely to contain a high proportion of functional sRNAs (including miRNAs). We also mapped the reads to available annotations, including annotated miRNAs, tRNAs and coding DNA sequences (CDSs), of the *B. terrestris* genome (Table S6). As expected, a high proportion of reads was incident to miRNAs (an average of 24.6% of redundant reads across all libraries, Table S6). The complexity of these reads was low, supporting the conclusion that a small number of sequences with high abundance were classified as miRNAs. However, we found that sample LW4 had a very low proportion of miRNAs compared to the other samples (< 5% miRNAs). A small proportion of reads, less than 2% in all samples, matched to tRNAs. Around 40% of the reads in each sample matched to annotated CDSs. The complexities of these reads were higher (> 0.1) and the proportion of non-redundant incident reads was consistently higher than the proportion of redundant reads, indicating that these reads were likely to have been degradation fragments (Table S6).

We next compared the similarity between replicates and treatments, for the mapped reads, using the Jaccard similarity index (Figure S2). The Jaccard index reflects the proportion of reads that are shared between samples (1.0 = samples identical, 0.0 = no shared reads) and was calculated for each pair of samples using the 500 most abundant reads in each sample[14]. In these comparisons, 15/16 libraries shared 50% or more of the 500 most highly expressed sequences between replicates of the same phenotype, the exceptional library being LW4 (Figure S2). Size class distribution plots showed that 22mer sRNA sequences were the most frequently expressed within each phenotype, again except for library LW4 (Figure S3).

Normalization of read abundances

We normalized the 16 libraries using the read count per total (rpt) normalization method[15-17], with the normalization total for each library set at 4 million, which was the median total read count (rounded up to the nearest million) of the accepted reads across all libraries (Table S4)[16]. To evaluate the outcome of the normalization, we plotted the distribution of pairwise differential expression, calculated using an offsetted fold change, across all read lengths[14].

For most samples, rpm normalization was appropriate. However, libraries EW4, LW4, EQ2 and LQ2 were significantly differentially expressed when compared to the other three replicates within each respective phenotype, particularly for reads of length 28-34 nt (Figure S4), which reduced the sequencing space for shorter fragments, introducing technical differential expression. LW4 also had a very low proportion of miRNAs (see above). We therefore excluded these libraries from further analysis.

MicroRNA prediction and differential expression analysis
Using the normalized matrix of abundances, we identified the *B. terrestris* miRNAs by aligning the sRNA-seq reads to the miRNAs listed under Hexapoda in *miRBase* v.21[12] allowing up to 2 mis-matches and accepting only reads aligning to the positive strand of the mature sequences (we thereby updated the set of predicted miRNAs for worker- destined larvae libraries EW1-4 and LW1–4) described in Sadd *et al*.[11]). A set of custom-made Perl scripts (available at https://github.com/mattlbeck/collins_et_al_MCDB) was used to create the set of conserved miRNAs. The abundances of mature miRNAs were calculated both at the read level and for mature miRNA variant regions following methods in Mohorianu *et al*.[14]. A miRNA variant region was defined by finding the per-nucleotide count coverage across all reads and libraries aligning to the mature miRNA. The largest increase and decrease in counts across the mature miRNA indicated the start and end of a variant region. The region count was calculated as the algebraic sum of read counts from reads incident to this region. These methods produced a final list of predicted miRNAs (Appendix 1).

To calculate differential expression between pairs of phenotypes, we determined confidence intervals from the replicates for each miRNA in each treatment as the range of the normalized read counts (i.e. difference between minimum and maximum normalized read counts)[18-21]. If, for a given miRNA, confidence intervals between two treatments did not overlap, the $\log_2$ offset fold change (OFC) was calculated from the ratio of the minimum expression level in the sample with higher mean expression and the maximum expression level in the sample with lower mean expression[18]. That is, for any sequence in samples *k* and *k'*, we defined the confidence intervals as $[min_k, max_k]$ for sample *k* and $[min_{k'}, max_{k'}]$ for sample *k'*, where min_k and $min_{k'}$ are the minimum values of the normalized expression levels in the replicates for the sequence, and max_k and $max_{k'}$ are the maximum values of the normalized expression levels in the replicates for the sequence. The $\log_2$ offset fold change was then calculated as follows:

For up-regulation (i.e. mean expression higher in sample *k'*), as

$$log_2 \frac{min_{k'}+o}{max_k+o};$$

and for down-regulation (i.e. mean expression higher in sample *k*), as

$$log_2 \frac{min_k+o}{max_{k'}+o}.$$

The offset (*o*), which was applied to each abundance before the fold change was calculated, was set at 20, thereby preventing the resulting rank of fold changes from being skewed by low-abundance reads[14].

Using these methods, we identified miRNAs that were differentially expressed between worker- and queen-destined larvae within instars (i.e. by comparing EW with EQ and LW

with LQ) and that were differentially expressed between instars within each caste trajectory (i.e. by comparing EQ with LQ and EW with LW). Following previous studies[8,22,23], we defined sequences as differentially expressed between phenotypes if the log_2 offset fold change was greater than one.

Northern blots

To verify the presence of miRNAs in the libraries prepared from cohort 1, and to validate the expression patterns identified by the bioinformatic analysis of these libraries, we used Northern blots to probe for miRNAs in RNA samples extracted from early- and late-instar queen- and worker-destined larvae in the ten selected colonies from cohort 2 (Table S5). We designed probes that were reverse-complementary to ten selected miRNAs (Table S7). These miRNAs fell into four classes: (a) six miRNAs (Bte-miR-13a, Bte-miR-87a, Bte-miR-100, Bte-miR-306, Bte-miR-6001-5p, Bte-miR-6001-3p) of the total of nine miRNAs identified (by bioinformatic analysis of our sRNA-seq data) as being differentially expressed between queen- and worker-destined larvae (Figure 1); note that Bte-miR-6001-5p and Bte-miR-6001-3p are part of the same duplex, but because both miRNAs showed differential expression and had a read count > 200, we treated the two arms as separate miRNAs; we omitted three miRNAs (Bte-miR-13-3p, Bte-miR-2518, and Bte-miR-8516) also identified as being differentially expressed between castes because they had relatively low expression levels compared to the majority of miRNAs (< 200 counts in any phenotype); (b) two miRNAs (Bte-miR-9a, Bte-miR-184) that were not differentially expressed between any of the four phenotypes but are homologues of miRNAs found by previous studies to be associated with larval or prepupal caste differences in *Apis mellifera* ([24-26]; Table S1); (c) one miRNA (Bte-miR-71) identified as being upregulated in early instar larvae compared to late instar larvae, and having a homologue that has previously been associated with caste differences in prepupae in *A. mellifera* ([24]; Table S1); and (d) one miRNA (Bte-miR-275) identified as being upregulated in late instar larvae compared to early instar larvae, and having a homologue that has previously been associated with caste differences in larvae in *A. mellifera* ([26]; Table S1).

For each selected miRNA, we produced at least two Northern blots (using RNA from each of two independent colony replicates) to compare the expression in the four phenotypes (EW, LW, EQ, LQ) alongside one another. We quantified the signal in each band using the imaging software *Quantity One* (Bio-Rad). The expression pattern shown by sRNA-seq was considered to be validated if each Northern blot showed the same expression pattern as that shown by sRNA-seq, i.e. either differential gene expression of more than two-fold in the same direction or no differential gene expression. In the case of Bte-miR-6001-3p, low expression levels meant that, in the five Northern blots attempted, only two yielded sufficient signal to allow us to determine relative expression.

We also used Northern blots of RNA extracted from larval and pupal samples in cohort 3 to investigate whether the caste-differentiated miRNAs that were validated by the Northern blots (Bte-miR-6001-5p and Bte-miR-6001-3p) were expressed in a tissue- or stage-specific manner.

To conduct Northern blots, we extracted total RNA from larvae sampled from cohort 2 using the Trizol protocol described above. We then separated the RNA on a 15% denaturing polyacrylamide gel and transferred the RNA to a Hybond-NX nylon membrane (GE Healthcare, Amersham, UK) using Semi-dry membrane transfer apparatus (Scie-plas, Cambridge, UK). We bound the RNA to the membrane by chemical crosslinking. We

hybridized membranes overnight at 37°C in UltraHyb-Oligo hybridization buffer (Ambion) with probes that were reverse complementary to the miRNA of interest and were labelled with 32P using T4 polynucleotide kinase and [γ^{-32} P] ATP. We rinsed the membranes with a wash solution (0.2 × sodium chloride/sodium citrate, 0.1% (w/v) sodium dodecyl sulphate (SDS)), then exposed them on a blank phospho-imaging screen inside a radioactive cassette (Fujifilm, Billingham, UK). We scanned the screens on a Molecular Imager FX Pro Plus (Bio-Rad, Hemel Hempsted, Hertfordshire) using *Quantity One* to visualize the signal. To allow membrane re-use, we stripped the membrane of its radioactive signal by incubating it in a stripping solution (pH 8.5, 0.1% SDS, 5mM EDTA) at 95°C for 20–40 minutes. All membranes were re-probed with U6, which is a small nuclear RNA acting as a loading control[27].

Identifying the genomic region and function of caste-differentiated miRNAs

To investigate miRNA function, we identified the region where validated caste-differentiated miRNAs were expressed on the *B. terrestris* genome[11]. We used *Basic Local Alignment Search Tool* (BLAST) software to identify the region, and the program *GBrowse* to identify the nucleotide sequence (and therefore the neighbouring genes) around each miRNA. These analyses revealed that the caste-associated miRNA, Bte-miR-6001, and its precursor sequence, form a mirtron[28,29] located in the gene *Very High Density Lipoprotein* (*VHDL*).

In addition, we used *miRanda*[30] to identify a list of targets for the two validated caste-associated miRNAs, Bte-miR-6001-5p and Bte-miR-6001-3p. We scanned the *B. terrestris* miRNAs against the 3'UTR regions of genes in *A. mellifera* on the NCBI database (http://www.ncbi.nlm.nih.gov/), since the 3'UTR regions are the sequences usually targeted by miRNAs[31-33] and the genome annotation in *A. mellifera*[34] is more extensive than in *B. terrestris*[11]. We then compared the 3'UTR region of these genes for sequence homology to *B. terrestris* genes, discarding sequences that were not conserved between *A. mellifera* and *B. terrestris*. We identified the gene ontologies of the remaining genes using the *Drosophila* gene ontology terms on FlyBase (http://flybase.org/).

Table S1. Expression patterns (from other studies) of selected miRNAs in female larvae or prepupae of *Apis mellifera*

miRNA	Pattern in *Apis mellifera*	References
Ame-miR-9a	More highly expressed in queen-destined prepupae than in worker-destined prepupae.	24
	More highly expressed in worker jelly than in royal jelly.	25
	More highly expressed in worker-destined larvae than in queen-destined larvae.	26
Ame-miR-184	More highly expressed in worker jelly than in royal jelly.	25
	Experimental increase caused queen-destined larvae to develop worker-like characteristics.	25
	More highly expressed in queen-destined larvae than in worker-destined larvae.	26
Ame-miR-71	More highly expressed in worker-destined prepupae than in queen-destined prepupae.	24
	More highly expressed in worker jelly than in royal jelly.	25
Ame-miR-275	More highly expressed in worker jelly than in royal jelly.	25
	More highly expressed in queen-destined larvae than in worker-destined larvae	26

Table S2. Details of samples of *Bombus terrestris* larvae used for sRNA-seq analysis (cohort 1). Columns of numerical data represent: total number of larvae that hatched during the period when brood development was being monitored by photographing colonies; of these, the number of early-instar larvae sampled (removed) and then pooled for RNA extraction; the number of early-instar larvae left within the colony; the number of late-instar larvae sampled and then pooled for RNA extraction; the number of late-instar larvae left within the colony to complete development to adulthood; and the adult caste of the remaining unsampled larvae, i.e. numbers of adult workers and queens eclosing. The far right column shows the sequencing library to which each colony contributed.

Colony	Queen status	Total number of larvae	Early-instar larvae sampled	Early-instar larvae left	Late-instar larvae sampled	Late-instar larvae left	Adult caste (unsampled larvae)	Experiment
QR-2	Queenright	85	41	44	19	25	25 workers<br>0 queens	Sequencing library EW1, LW1
QR-7	Queenright	105	44	61	21	40	40 workers<br>0 queens	Sequencing library EW2, LW2
QR-15	Queenright	111	43	68	23	45	45 workers<br>0 queens	Sequencing library EW3, LW3
QR-17	Queenright	96	36	60	22	38	38 workers<br>0 queens	Sequencing library EW3, LW4
**Sub-total**	**Queenright**	**397**	**164**	**237**	**85**	**148**	**148 workers<br>0 queens**	**4 Sequencing worker libraries**
QL-10	Queenless	69	23	46	22	24	0 workers<br>24 queens	Sequencing library EQ1, LQ1
QL-13	Queenless	20	10	10	5	5	0 workers<br>5 queens	Sequencing library EQ2, LQ2
QL-14	Queenless	65	25	33	27	13	2 workers<br>11 queens	Sequencing library EQ3, LQ3
QL-19	Queenless	38	18	20	11	9	0 workers<br>9 queens	Sequencing library EQ4, LQ4
**Sub-total**	**Queenless**	**192**	**76**	**109**	**65**	**51**	**2 workers<br>49 queens**	**4 Sequencing queen libraries**

Table S3. Details of samples of *Bombus terrestris* larvae used for Northern blot analysis (cohort 2). Columns of numerical data represent: total number of larvae that hatched during the period when brood development was being monitored by photographing colonies; of these, the number of early-instar larvae sampled (removed) and then pooled for RNA extraction; the number of early-instar larvae left within the colony; the number of late-instar larvae sampled and then pooled for RNA extraction; the number of late-instar larvae left within the colony to complete development to adulthood; and the adult caste of the remaining unsampled larvae, i.e. numbers of adult workers and queens eclosing. The far right column shows the Northern blot sample to which each colony contributed.

Colony	Queen status	Total number of larvae	Early-instar larvae sampled	Early-instar larvae left	Late-instar larvae sampled	Late-instar larvae left	Adult caste (unsampled larvae)	Experiment
QR-21	Queenright	137	59	78	20	58	58 workers<br>0 queens	Northern blot NB.EW1, NB.LW1
QR-22	Queenright	95	43	52	6	46	45 workers<br>0 queens<br>1 male	Northern blot NB.EW2, NB.LW2
QR-23	Queenright	104	40	64	22	42	42 workers<br>0 queens	Northern blot NB.EW3, NB.LW3
QR-24	Queenright	125	47	78	25	53	53 workers<br>0 queens	Northern blot NB.EW4, NB.LW4
QR-25	Queenright	90	34	56	19	37	37 workers<br>0 queens	Northern blot NB.EW5, NB.LW5
**Sub-total**	**Queenright**	**551**	**223**	**328**	**92**	**236**	**235 workers**<br>**0 queens**<br>**1 male**	**5 Northern blot worker samples**
QR-23	Queenless	81	33	48	17	31	1 worker<br>30 queens	Northern blot NB.EQ1, NB.LQ1
QR-27	Queenless	63	31	32	11	21	0 workers<br>21 queens	Northern blot NB.EQ2, NB.LQ2
QR-29	Queenless	79	30	49	10	39	0 workers<br>39 queens	Northern blot NB.EQ3, NB.LQ3
QR-32	Queenless	26	11	15	10	5	1 worker<br>4 queens	Northern blot NB.EQ4, NB.LQ4
QR-33	Queenless	35	17	18	8	10	1 worker<br>9 queens	Northern blot NB.EQ5, NB.LQ5
**Sub-total**	**Queenless**	**284**	**122**	**162**	**56**	**106**	**3 workers**<br>**103 queens**	**5 Northern blot queen samples**

Table S4. Details of libraries prepared from samples of *Bombus terrestris* larvae and used for sRNA-seq (cohort 1). Columns of numerical data represent: number of larvae pooled to make each library; total read count (total number of sequence reads obtained); filtered reads, %filt (number and percentage of reads that do not contain unassigned nucleotides); reads with an adapter sequence, %adp (number and percentage of reads for which the first 8 nt of the 3' adapter was found); accepted reads, %acc (number and percentage of accepted reads (longer than 16nt) after the HD signature was trimmed); NR (number of non-redundant (unique) reads in each sample); C (sequence complexity, i.e. ratio of non-redundant reads to redundant reads (all reads) in each sample); GMR, %GMR (number and percentage of redundant reads mapping to genome); GMNR, %GMNR (number and percentage of non-redundant reads mapping to genome); and GMC (complexity of reads mapping to genome).

Library	Colony	Number of larvae pooled	Total read count	Filtered reads	%filt	With Adapter	%adp	Accepted reads	%acc	NR	C	GMR	% GMR	GMNR	% GMNR	GMC
EW1	QR-2	41	5,669,480	5,664,018	99.9%	4,779,769	84.4%	4,389,555	91.8%	636,541	0.145	3,036,380	69.2%	373,719	58.7%	0.123
EW2	QR-7	44	7,029,923	7,023,107	99.9%	5,843,873	83.2%	5,228,691	89.5%	707,881	0.135	3,530,423	67.5%	395,264	55.8%	0.112
EW3	QR-15	43	2,996,910	2,993,960	99.9%	2,541,747	84.9%	2,183,683	85.9%	357,548	0.164	1,498,610	68.6%	198,877	55.6%	0.133
EW4	QR-17	36	3,950,811	3,947,018	99.9%	3,141,459	79.6%	2,640,102	84.0%	421,203	0.160	1,736,816	65.8%	226,845	53.9%	0.131
LW1	QR-2	19	4,101,572	4,097,537	99.9%	3,607,147	88.0%	2,791,657	77.4%	308,282	0.110	1,788,320	64.1%	153,655	49.8%	0.086
LW2	QR-7	21	3,934,876	3,931,076	99.9%	3,425,729	87.1%	2,587,881	75.5%	262,454	0.101	1,581,384	61.1%	121,744	46.4%	0.077
LW3	QR-15	23	4,659,419	4,654,950	99.9%	3,173,336	68.2%	2,173,379	68.5%	236,803	0.109	1,171,029	53.9%	100,411	42.4%	0.086
LW4	QR-17	22	5,506,360	5,500,855	99.9%	3,763,985	68.4%	2,887,785	76.7%	126,551	0.044	2,153,291	74.6%	77,645	61.4%	0.036
EQ1	QL-10	23	5,448,946	5,443,661	99.9%	4,215,116	77.4%	3,745,119	88.8%	386,372	0.103	2,904,990	77.6%	240,694	62.3%	0.083
EQ2	QL-14	10	6,847,581	6,840,897	99.9%	6,071,848	88.8%	5,176,221	85.2%	405,094	0.078	4,054,667	78.3%	260,220	64.2%	0.064
EQ3	QL-15	25	5,530,990	5,525,744	99.9%	4,546,294	82.3%	4,098,251	90.1%	367,349	0.090	2,823,814	68.9%	202,938	55.2%	0.072
EQ4	QL-19	18	5,765,081	5,759,588	99.9%	4,933,609	85.7%	4,462,165	90.4%	268,380	0.060	3,619,609	81.1%	175,293	65.3%	0.048
LQ1	QL-10	22	5,097,236	5,092,279	99.9%	4,017,539	78.9%	3,004,010	74.8%	245,888	0.082	2,193,319	73.0%	130,870	53.2%	0.060
LQ2	QL-14	5	9,661,348	9,651,974	99.9%	6,883,877	71.3%	5,953,735	86.5%	293,502	0.049	4,418,805	74.2%	162,568	55.4%	0.037
LQ3	QL-15	27	5,993,360	5,987,608	99.9%	4,298,974	71.8%	3,430,445	79.8%	267,942	0.078	2,454,929	71.6%	140,486	52.4%	0.057
LQ4	QL-19	11	3,401,702	3,398,524	99.9%	2,735,155	80.5%	2,005,763	73.3%	211,474	0.105	1,214,414	60.5%	98,002	46.3%	0.081

Table S5. Details of samples prepared from pooled *Bombus terrestris* larvae and used for Northern blots (cohort 2).

Sample name	Colony	Number of larvae pooled	Description
NB.EW1	QR-21	59	Early-instar worker-destined RNA sample
NB.EW2	QR-22	43	Early-instar worker-destined RNA sample
NB.EW3	QR-23	40	Early-instar worker-destined RNA sample
NB.EW4	QR-24	47	Early-instar worker-destined RNA sample
NB.EW5	QR-25	34	Early-instar worker-destined RNA sample
NB.LW1	QR-21	20	Late-instar worker-destined RNA sample
NB.LW2	QR-22	6	Late-instar worker-destined RNA sample
NB.LW3	QR-23	22	Late-instar worker-destined RNA sample
NB.LW4	QR-24	25	Late-instar worker-destined RNA sample
NB.LW5	QR-25	19	Late-instar worker-destined RNA sample
NB.EQ1	QL-23	33	Early-instar queen-destined RNA sample
NB.EQ2	QL-27	31	Early-instar queen-destined RNA sample
NB.EQ3	QL-29	30	Early-instar queen-destined RNA sample
NB.EQ4	QL-32	11	Early-instar queen-destined RNA sample
NB.EQ5	QL-33	17	Early-instar queen-destined RNA sample
NB.LQ1	QL-23	17	Late-instar queen-destined RNA sample
NB.LQ2	QL-27	11	Late-instar queen-destined RNA sample
NB.LQ3	QL-29	10	Late-instar queen-destined RNA sample
NB.LQ4	QL-32	10	Late-instar queen-destined RNA sample
NB.LQ5	QL-33	8	Late-instar queen-destined RNA sample

Table S6. Details of the numbers and percentages of miRNAs, tRNAs, and coding DNA sequences (CDSs) represented in sRNA-seq data from samples of *Bombus terrestris* larvae. Columns of numerical data represent, for each class of gene: R, %R (the number and percentage (of the total sequences that mapped to the genome) that were redundant reads (all reads)); NR, %NR (the number and percentage (of the total sequences that mapped to the genome) that were non-redundant reads (unique reads)); and C (the sequence complexity, i.e. ratio of non-redundant reads to redundant reads (all reads) in each sample).

Library	miRNA					tRNA					CDS				
	R	%R	NR	%NR	C	R	%R	NR	%NR	C	R	%R	NR	%NR	C
EW1	961,452	31.7	3,178	0.9	0.003	33,008	1.1	5,597	1.5	0.170	1,208,311	39.8	210,878	56.4	0.175
EW2	1,021,753	28.9	3,497	0.9	0.003	43,891	1.2	6,129	1.6	0.140	1,362,923	38.6	226,372	57.3	0.166
EW3	415,231	27.7	2,200	1.1	0.005	15,720	1.0	3,581	1.8	0.228	543,422	36.3	108,404	54.5	0.199
EW4	538,668	31.0	2,330	1.0	0.004	26,341	1.5	3,708	1.6	0.141	672,984	38.7	121,152	53.4	0.180
LW1	480,675	26.9	2,309	1.5	0.005	15,788	0.9	2,040	1.3	0.129	725,295	40.6	95,234	62.0	0.131
LW2	278,507	17.6	1,917	1.6	0.007	6,790	0.4	1,072	0.9	0.158	586,591	37.1	78,745	64.7	0.134
LW3	287,394	24.5	1,702	1.7	0.006	15,592	1.3	1,830	1.8	0.117	450,082	38.4	59,820	59.6	0.133
LW4	99,306	4.6	874	1.1	0.009	2,744	0.1	351	0.5	0.128	740,532	34.4	53,908	69.4	0.073
EQ1	761,959	26.2	2,819	1.2	0.004	21,271	0.7	2,156	0.9	0.101	1,133,082	39.0	139,509	58.0	0.123
EQ2	705,579	17.4	2,658	1.0	0.004	18,493	0.5	2,152	0.8	0.116	1,313,675	32.4	160,748	61.8	0.122
EQ3	765,431	27.1	2,704	1.3	0.004	18,731	0.7	1,661	0.8	0.089	1,242,250	44.0	118,877	58.6	0.096
EQ4	902,365	24.9	2,918	1.7	0.003	9,995	0.3	971	0.6	0.097	1,507,663	41.7	107,387	61.3	0.071
LQ1	499,825	22.8	2,171	1.7	0.004	19,247	0.9	1,457	1.1	0.076	767,063	35.0	76,331	58.3	0.100
LQ2	1,441,232	32.6	3,089	1.9	0.002	62,125	1.4	1,728	1.1	0.028	2,070,890	46.9	92,604	57.0	0.045
LQ3	691,948	28.2	2,547	1.8	0.004	22,420	0.9	1,816	1.3	0.081	969,125	39.5	81,395	57.9	0.084
LQ4	279,908	23.0	1,929	2.0	0.007	6,268	0.5	894	0.9	0.143	500,492	41.2	60,504	61.7	0.121

Table S7. MiRNAs from *Bombus terrestris* female larvae probed using Northern blots, their mature sequences (predicted from the *B. terrestris* genome [11]) and the corresponding probe sequences, along with corresponding sequences for the U6 control.

miRNA name	Mature miRNA sequence	Probe sequence
miR-13	TATCACAGCCATTTTGTTGACT	AGTCAACAAAATGGCTGTGATA
miR-87a	GTGAGCAAAGTTTCAGGTGTGT	ACACACCTGAAACTTTGCTCAC
miR-100	AACCCGTAGATCCGAACTTGTG	CACAAGTTCGGATCTACGGGTT
miR-306	TCAGGTACTGAGTGACTCTGAG	CTCAGAGTCACTCAGTACCTGA
miR-6001-5p	GUAGGUAACGACUGAUGGGAACA	TGTTCCCATCAGTCGTTACCTAC
miR-6001-3p	UUCUCUUUGGUUGUUACCACUA	TAGTGGTAACAACCAAAGAGAA
miR-9a	UCUUUGGUUAUCUAGCUGUAUGA	TCATACAGCTAGATAACCAAAGA
miR-184	UGGACGGAGAACUGAUAAGG	CCTTATCAGTTCTCCGTCCA
miR-71	UGAAAGACAUGGGUAGUGAGAUG	CATCTCACTACCCATGTCTTTCA
miR-275	UCAGGUACCUGAAGUAGCGCGCG	CGCGCGCTACTTCAGGTACCTGA
U6 control	GGAACGAUACAGAGAAGAUUAGC	GCTAATCTTCTCTGTATCGTTCC

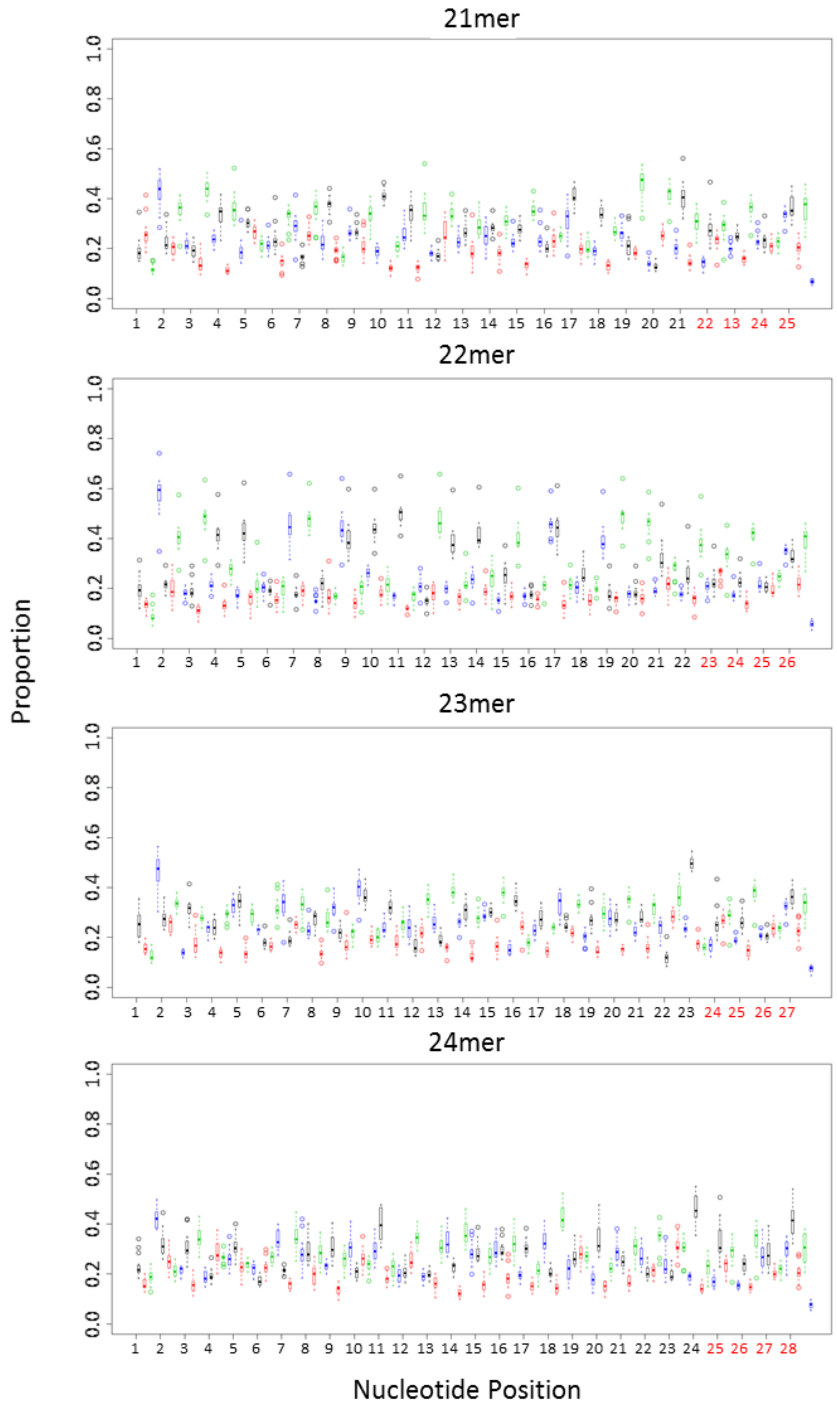

Figure S1: Boxplots showing the distribution of proportions of each specific nucleotide base at any given position in the 21mer, 22mer, 23mer, and 24mer sequences identified through sRNAseq in *Bombus terrestris*, including the four-nucleotide signature of the 5' end of the 3' HD adapter sequence (positions highlighted in red). X axis, the nucleotide position on the focal sequence; Y axis, the proportion of each base (black represents adenine, red represents cytosine, green represents guanine, blue represents thymine) at a given nucleotide position on the focal sequence. In each boxplot, the thick horizontal bar is the median, box edges are quartiles, whiskers are the upper and lower 5%, and circles are outliers. Data from the 12 libraries remaining in the final analysis.

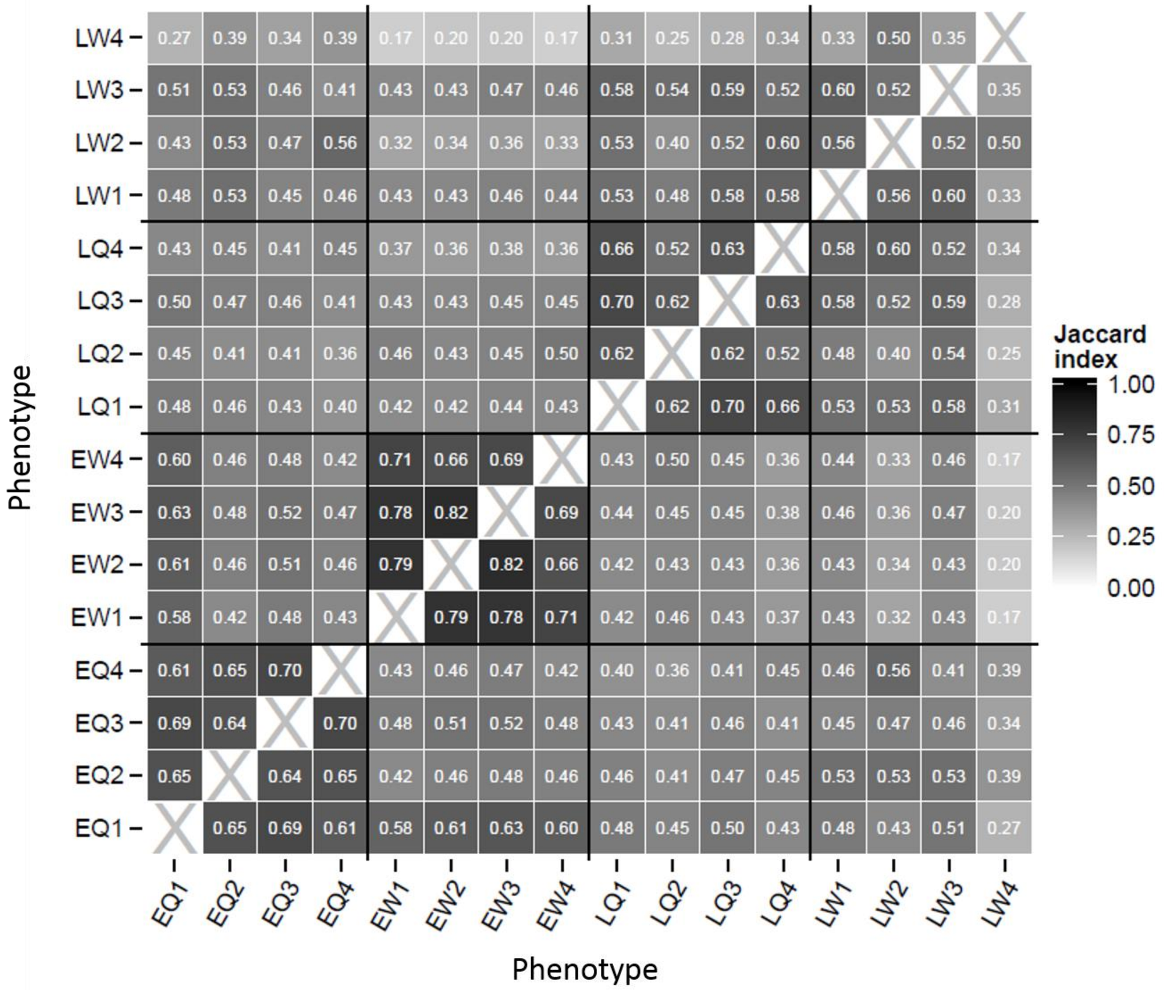

Figure S2. Matrix of Jaccard indices comparing 16 sRNA-seq libraries prepared from *Bombus terrestris* female larvae from cohort 1. For the 500 most abundant sequences, the Jaccard index denotes the proportion of shared sequences between pairs of libraries. There were four biological replicates of each of four phenotypes, the phenotypes being early-instar queen-destined larvae (EQ), early-instar worker-destined larvae (EW), late-instar queen-destined larvae (LQ) and late-instar worker-destined larvae (LW), the suffixed numbers representing replicate numbers.

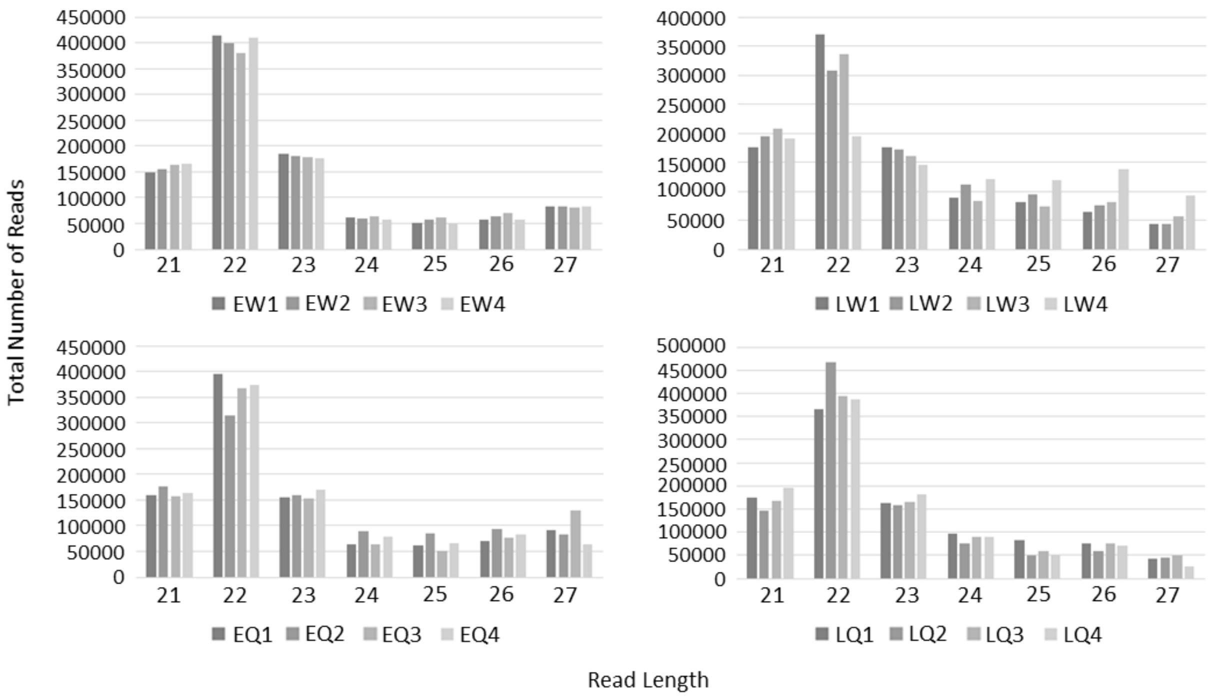

Figure S3. Size class distribution plots (following normalization) of RNA sequences (of lengths 21-27 nt) from the 16 sRNA-seq libraries prepared from *Bombus terrestris* female larvae (four biological replicates of each of the four phenotypes, EW, EQ, LW and LQ, as defined in the legend to Figure S2).

Early Worker libraries

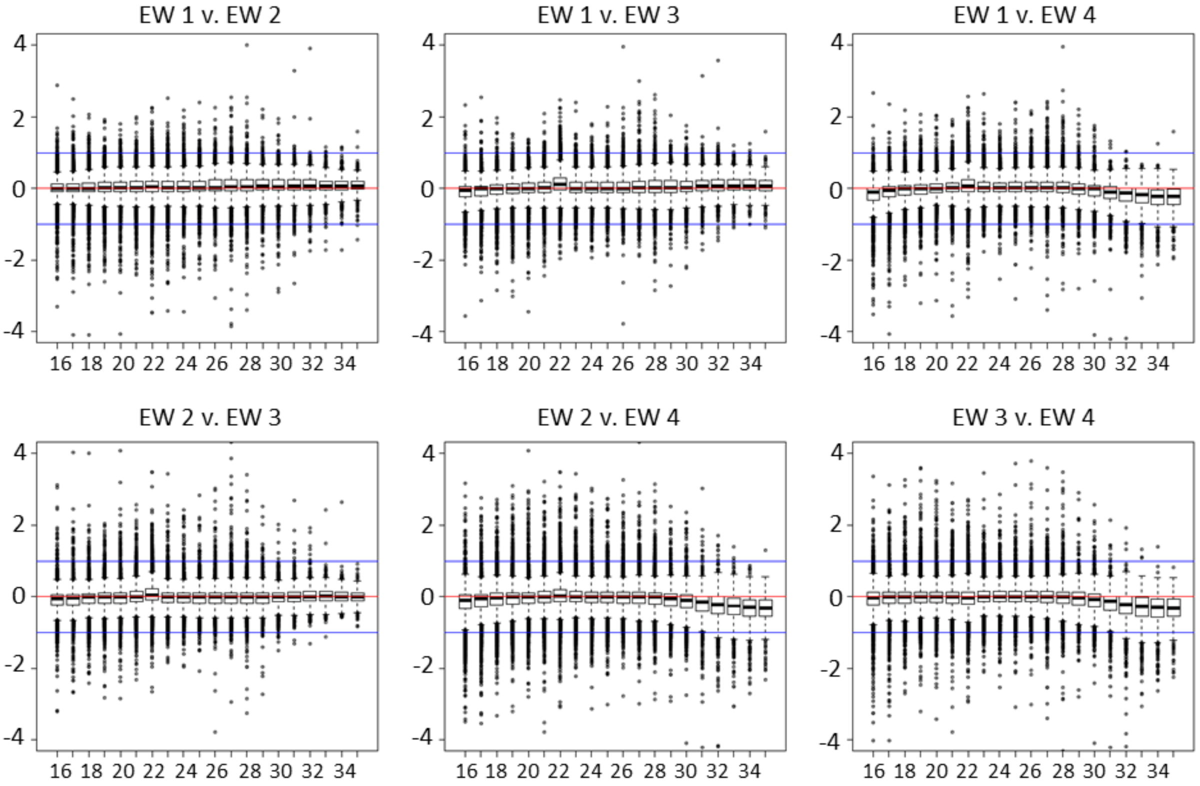

Late Worker libraries

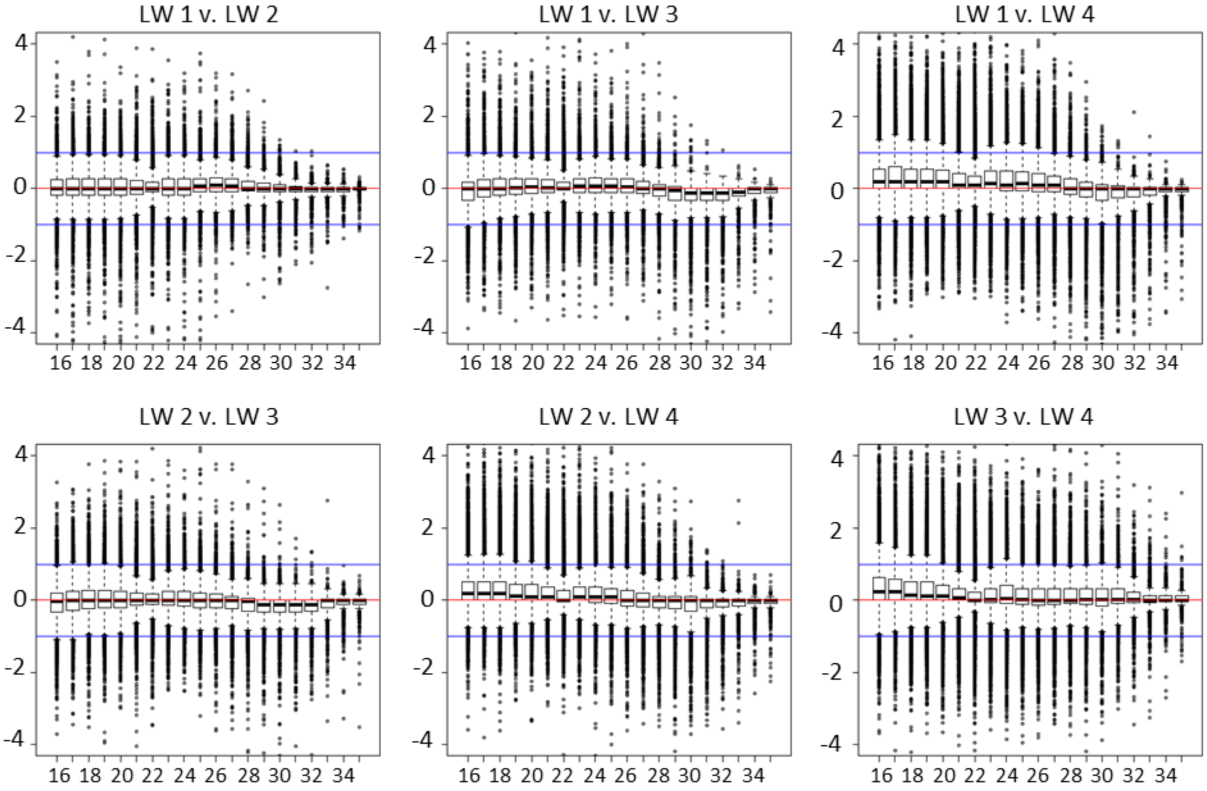

Early Queen libraries

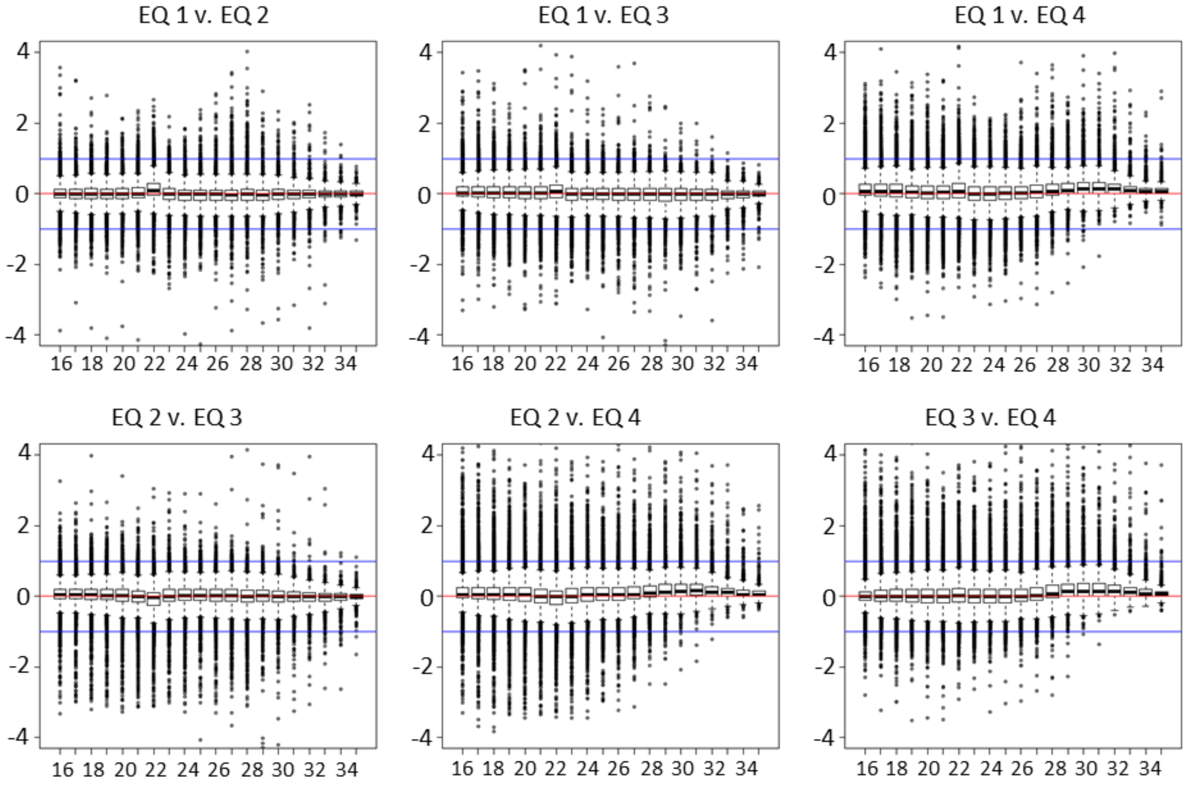

Late Queen libraries

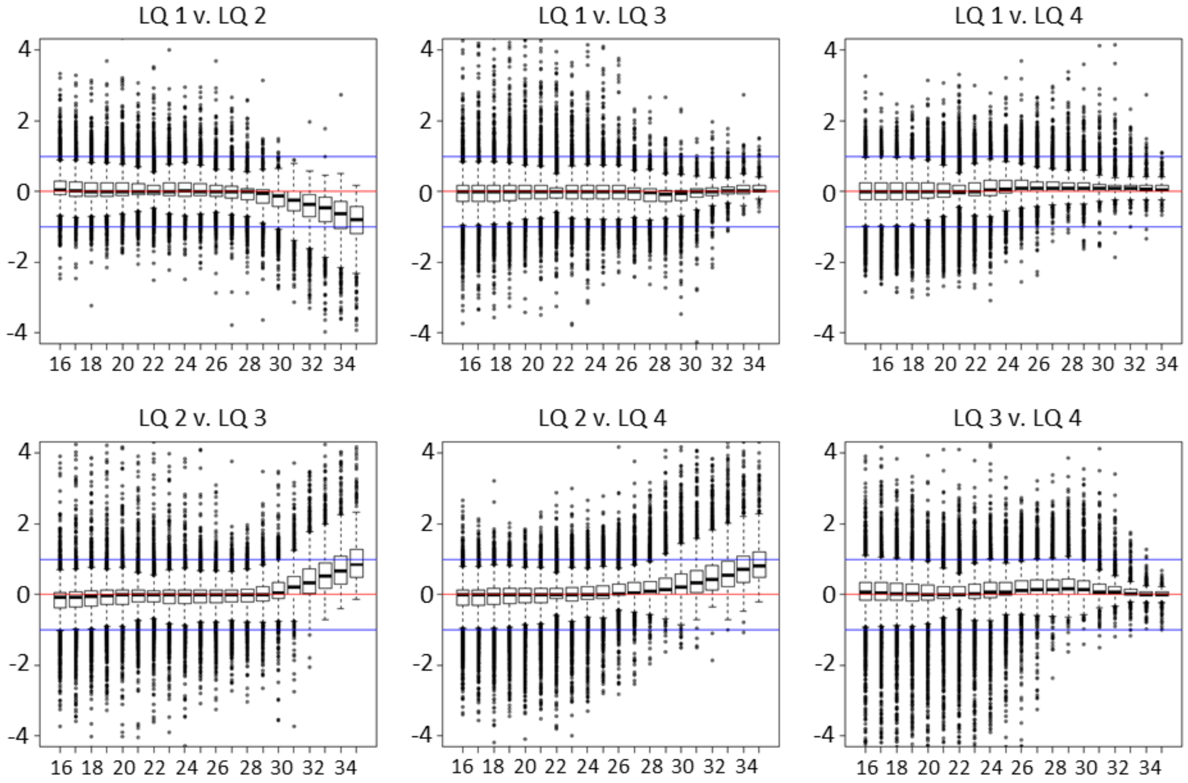

Figure S4. Distribution of differences in normalized gene expression levels (offset fold change) between pairs of biological replicates within each phenotype class in 16 sRNA-seq libraries (four phenotype classes, each with four biological replicates) prepared from *Bombus terrestris* female larvae, for reads within the size-class range, 16-35 nt. Figure panel header: replicates being compared, with phenotypes as defined in legend of Figure S2; X axis, size class of reads being compared; Y axis, $\log_2$ fold change (OFC) value following normalization. The red line at Y = 0 denotes no differential expression between replicates. The blue lines correspond to ± $0.5\log_2$(OFC), which approximates ± 1.5(OFC). In each boxplot, the thick horizontal bar is the median, box edges are quartiles, whiskers are the upper and lower 5%, and black points are outliers.

References cited in Supplementary Information

1 Duchateau, M. J. & Velthuis, H. H. W. Development and reproductive strategies in *Bombus* colonies. *Behaviour* **107**, 186-207 (1988).

2 Holland, J. G., Guidat, F. S. & Bourke, A. F. G. Queen control of a key life-history event in a eusocial insect. *Biol. Letters* **9**, 20130056 (2013).

3 Cnaani, J., Borst, D. W., Huang, Z. Y., Robinson, G. E. & Hefetz, A. Caste determination in *Bombus terrestris*: differences in development and rates of JH biosynthesis between queen and worker larvae. *J. Insect Physiol.* **43**, 373-381 (1997).

4 Pereboom, J. J. M., Jordan, W. C., Sumner, S., Hammond, R. L. & Bourke, A. F. G. Differential gene expression in queen-worker caste determination in bumble bees. *Proc. Roy. Soc. B-Biol. Sci.* **272**, 1145-1152 (2005).

5 Lopez-Vaamonde, C. *et al.* Effect of the queen on worker reproduction and new queen production in the bumble bee *Bombus terrestris*. *Apidologie* **38**, 171-180 (2007).

6 Bourke, A. F. G. & Ratnieks, F. L. W. Kin-selected conflict in the bumble bee *Bombus terrestris* (Hymenoptera : Apidae). *Proc. Roy. Soc. B-Biol. Sci.* **268**, 347-355 (2001).

7 Cnaani, J., Robinson, G. E. & Hefetz, A. The critical period for caste determination in *Bombus terrestris* and its juvenile hormone correlates. *J. Comp. Physiol. A* **186**, 1089-1094 (2000).

8 Feldmeyer, B., Elsner, D. & Foitzik, S. Gene expression patterns associated with caste and reproductive status in ants: worker-specific genes are more derived than queen-specific ones. *Mol. Ecol.* **23**, 151-161 (2014).

9 Sorefan, K. *et al.* Reducing ligation bias of small RNAs in libraries for next generation sequencing. *Silence* **3**, 4 (2012).

10 Yoshikawa, T., Sanders, A. R. & DeteraWadleigh, S. D. Contamination of sequence databases with adaptor sequences. *Am. J. Hum. Genet.* **60**, 463-466 (1997).

11 Sadd, B. M. *et al.* The genomes of two key bumblebee species with primitive eusocial organization. *Genome Biol.* **16**, 76 (2015).

12 Kozomara, A. & Griffiths-Jones, S. miRBase: integrating microRNA annotation and deep-sequencing data. *Nucleic Acids Res.* **39**, D152-D157 (2011).

13 Prufer, K. *et al.* PatMaN: rapid alignment of short sequences to large databases. *Bioinformatics* **24**, 1530-1531 (2008).

14 Mohorianu, I. *et al.* Profiling of short RNAs during fleshy fruit development reveals stage-specific sRNAome expression patterns. *Plant J.* **67**, 232-246 (2011).

15 Mortazavi, A., Williams, B. A., Mccue, K., Schaeffer, L. & Wold, B. Mapping and quantifying mammalian transcriptomes by RNA-Seq. *Nat. Methods* **5**, 621-628 (2008).

16 McCormick, K. P., Willman, M. R. & Meyers, B. C. Experimental design, preprocessing, normalization and differential expression analysis of small RNA sequencing experiments. *Silence* **2**, 2 (2011).

17 Dillies, M. A. *et al.* A comprehensive evaluation of normalization methods for Illumina high-throughput RNA sequencing data analysis. *Brief Bioinform.* **14**, 671-683 (2013).

18 Mohorianu, I., Stocks, M. B., Wood, J., Dalmay, T. & Moulton, V. CoLIde A bioinformatics tool for CO-expression based small RNA Loci Identification using high-throughput sequencing data. *RNA Biol.* **10**, 1221-1230 (2013).

19 Morey, J. S., Ryan, J. C., Van Dolah, F. M. Microarray validation: factors influencing correlation between oligonucleotide microarrays and real-time PCR. *Biol. Proced. Online* **8**, 175-193 (2006).

20 Soneson, C., Delorenzi, M. A comparison of methods for differential expression analysis of RNA-seq data. *BMC Bioinform.* **14**, 91 (2013).

21 Rapaport, F., Khanin, R., Liang, Y., Pirun, M., Krek, A., Zumbo, P., Mason, C. E., Socci, N.D., Betel, D. Comprehensive evaluation of differential gene expression analysis methods for RNA-seq data. *Genome Biol.* **14**, R95 (2013).

22 Lopez-Gomollon, S., Mohorianu, I., Szittya, G., Moulton, V. & Dalmay, T. Diverse correlation patterns between microRNAs and their targets during tomato fruit development indicates different modes of microRNA actions. *Planta* **236**, 1875-1887 (2012).

23 Ferreira, P. G. *et al.* Transcriptome analyses of primitively eusocial wasps reveal novel insights into the evolution of sociality and the origin of alternative phenotypes. *Genome Biol.* **14**, R20 (2013).

24 Weaver, D. B. *et al.* Computational and transcriptional evidence for microRNAs in the honey bee genome. *Genome Biol.* **8**, 97 (2007).

25 Guo, X. Q. *et al.* Recipe for a busy bee: microRNAs in honey bee caste determination. *PLOS One* **8**, e81661 (2013).

26 Shi, Y. Y., Zheng, H. J., Pan, Q. Z., Wang, Z. L. & Zeng, Z. J. Differentially expressed microRNAs between queen and worker larvae of the honey bee (*Apis mellifera*). *Apidologie* **46**, 35-45 (2015).

27 Lopez-Gomollon, S. Detecting sRNAs by Northern blotting. In: *MicroRNAs In Development: Methods And Protocols* (ed. Dalmay, T.) 25-38 (Humana Press, 2011).

28 Okamura, K., Hagen, J. W., Duan, H., Tyler, D. M. & Lai, E. C. The mirtron pathway generates microRNA-class regulatory RNAs in *Drosophila*. *Cell* **130**, 89-100 (2007).

29 Axtell, M. J., Westholm, J. O. & Lai, E. C. Vive la difference: biogenesis and evolution of microRNAs in plants and animals. *Genome Biol.* **12**, 221 (2011).

30 Enright, A. J. *et al.* MicroRNA targets in Drosophila. *Genome Biol.* **5**, R1 (2004).

31 Bartel, D. P. MicroRNAs: genomics, biogenesis, mechanism, and function. Cell 116, 281-297 (2004).

32 Lytle, J. R., Yario, T. A. & Steitz, J. A. Target mRNAs are repressed as efficiently by microRNA-binding sites in the 5 ' UTR as in the 3 ' UTR. *Proc. Natl Acad. Sci. USA* **104**, 9667-9672 (2007).

33 Fang, Z. & Rajewsky, N. The impact of miRNA target sites in coding sequences and in 3'UTRs. *PLOS One* **6**, e18067 (2011).

34 Elsik, C. G. *et al.* Finding the missing honey bee genes: lessons learned from a genome upgrade. *BMC Genomics* **15**, 86 (2014).
